# Survey and Synthesis of State of the Art in Driver Monitoring

**DOI:** 10.3390/s21165558

**Published:** 2021-08-18

**Authors:** Anaïs Halin, Jacques G. Verly, Marc Van Droogenbroeck

**Affiliations:** Department of Electrical Engineering and Computer Science, University of Liège, B-4000 Liège, Belgium; jacques.verly@uliege.be (J.G.V.); m.vandroogenbroeck@uliege.be (M.V.D.)

**Keywords:** survey, driver monitoring, driver state, sensor, indicator, drowsiness, mental workload, distraction, emotions, under the influence

## Abstract

Road vehicle accidents are mostly due to human errors, and many such accidents could be avoided by continuously monitoring the driver. Driver monitoring (DM) is a topic of growing interest in the automotive industry, and it will remain relevant for all vehicles that are not fully autonomous, and thus for decades for the average vehicle owner. The present paper focuses on the first step of DM, which consists of characterizing the state of the driver. Since DM will be increasingly linked to driving automation (DA), this paper presents a clear view of the role of DM at each of the six SAE levels of DA. This paper surveys the state of the art of DM, and then synthesizes it, providing a unique, structured, polychotomous view of the many characterization techniques of DM. Informed by the survey, the paper characterizes the driver state along the five main dimensions—called here “(sub)states”—of drowsiness, mental workload, distraction, emotions, and under the influence. The polychotomous view of DM is presented through a pair of interlocked tables that relate these states to their indicators (e.g., the eye-blink rate) and the sensors that can access each of these indicators (e.g., a camera). The tables factor in not only the effects linked directly to the driver, but also those linked to the (driven) vehicle and the (driving) environment. They show, at a glance, to concerned researchers, equipment providers, and vehicle manufacturers (1) most of the options they have to implement various forms of advanced DM systems, and (2) fruitful areas for further research and innovation.

## 1. Introduction

A report published in 2018 [1] provides the results of an analysis performed on data about the events and related factors that led to crashes of small road vehicles from 2005 to 2007 across the USA. It indicates that the critical reasons for these crashes are likely attributable to the driver (in 94% of the cases), the vehicle (2%), the environment (2%), and unknown causes (2%). An overwhelming proportion of these crashes is thus due to human error. It is widely recognized that most of them could be avoided by constantly monitoring the driver [2,3], and by taking proper, timely actions when necessary.

Monitoring the driver is thus critically important, and this applies to all vehicles, with the exception of those that are fully autonomous, that is, where the driver does not control the vehicle under any circumstances. Given that the average driver will not own a fully-autonomous vehicle for decades to come, “driver monitoring (DM) ” will remain critically important during all this time. Note that the list of all abbreviations and their definitions appears after Section 11, before the appendices.

This paper focuses on the topic of DM, which is usefully viewed as consisting of two successive steps. In the first, one characterizes the driver, or more precisely the state of the driver, and, in the second, one decides what safety actions to take based on this characterization. For example, in the monitoring of drowsiness, the first step might compute the level of drowsiness, whereas the second might check whether this level is at, or will soon reach, a critical level. More generally, the decision process should ideally fuse the various characterization parameters available and predict the future state of the driver based on them. This paper focuses almost exclusively on the characterization of the state of the driver, that is, on the first step in DM, which is also the one that is almost exclusively considered in the literature.

By “state of the driver” or “driver state”, we mean, in a loose way, the state or situation that the driver is in from various perspectives, in particular physical, physiological, psychological, and behavioral. To deal with this driver state in a manageable, modular way, we consider a specific number of distinct facets (such as drowsiness) of this driver state, which we call “driver (sub)states”. In the sequel, “state” thus refers either to the global state of the driver or to one of its facets, or substates. This paper covers the main (sub)states of drowsiness, mental workload, distraction, emotions, and under the influence, which emerge as being the most significant ones in the literature.

The core of the paper focuses on the characterization of each of these (sub)states, using indicators (of this state) and sensors (to access the values of these indicators in real time and in real driving conditions). In the example of the (sub)state of drowsiness, an indicator thereof is the eye-blink rate, and it can be accessed using a camera.

DM is important, whether the vehicle is equipped with some form of “driving automation (DA)” (except for full automation) or not. In future vehicles, DA and DM will need to increasingly interact, and they will need to be designed and implemented in a synergistic way. While the paper focuses on DM (and, more precisely, on its characterization part), it considers and describes, at a high-level, how DM and DA interact at the various, standard levels of DA.

As suggested by its title, the paper comprises two main phases: (1) it reports on a systematic survey of the state of the art of DM (as of early 2021); (2) it provides a synthesis of the many characterization techniques of DM. This synthesis leads to an innovative, structured, polychotomous view of the recent developments in the characterization part of DM. In a nutshell, this view is provided by two interlocked tables that involve the main driver (sub)states, the indicators of these states, and the sensors allowing access to the values of these indicators. The polychotomy presented should prove useful to researchers, equipment providers, and vehicle manufacturers for organizing their approach concerning the characterization and monitoring of the state of the driver.

Section 2 describes the standard levels of DA, and the role played by DM for each. Section 3 indicates the strategy for, and the results of, our survey of the literature on DM. Section 4 describes the rationale and strategy for expressing the characterization of the driver state as much as possible in terms of the triad of the (sub)states, indicators, and sensors. Section 5 provides our innovative, structured, polychotomous view of the characterization part of DM. Section 6, Section 7, Section 8, Section 9 and Section 10 successively describe the five driver (sub)states that the survey revealed as being the most important. Section 11 summarizes and concludes.

## 2. Driving Automation and Driver Monitoring

In autonomous vehicles—also called self-driving or fully-automated vehicles—DM plays a critical role as long as the automation allows the driver to have some control over the vehicle. This section describes the interaction between DM and DA in the context of the six levels of DA defined by the Society of Automotive Engineers (SAE) International [4], ranging from zero (no automation) to five (full automation).

Table 1, inspired by the SAE J3016 Levels of Driving Automation Graphic, describes the role of each of the three key actors in the driving task, namely the driver, the driver-support (DS) features, and the automated-driving (AD) features, at each of the six SAE levels. We also integrated into this table a fourth actor, that is, DM, as its role is crucial at all levels except the highest, to ensure that the state of the driver allows him/her to perform the driving task safely, when applicable. Throughout, we use the inclusive pronoun “he/she” and adjective “his/her” to refer to the driver.

We now discuss some terminology. In Section 1, we introduced the term “driving automation (DA)” (as a convenient, companion term for DM) and, in the previous paragraph, the SAE-suggested term “automated driving (AD)”. While these two terms seem to further add to a jumble of terms and abbreviations, they both appear in the literature through their corresponding systems, that is, the “driving-automation system (DAS)” and “automated-driving system (ADS)”. An ADS is a system consisting of the AD features, and a DAS is a system that includes, among other things, both DS features and AD features. One could also view the DS features as constituting a system, but this is not needed here.

In future vehicles with progressively increasing degrees of automation, the development of DASs and, in particular, of ADSs should go hand-in-hand with the development of driver-monitoring systems (DMSs). The next four paragraphs complement the information in Table 1.

At Levels 0 to 2, the driver is responsible for the driving task, and he/she may be aided by a variable number of DS features such as automatic emergency braking, adaptive cruise control, and lane centering. At Level 1, the DS features execute the subtask of controlling either the lateral motion or the longitudinal motion of the vehicle (but not both), expecting the driver to perform the rest of the driving task. At Level 2, the DS features execute the subtasks of controlling both the lateral motion and the longitudinal motion, expecting the driver to complete the object-and-event-detection-and-response (OEDR) subtask and to supervise these features. At Levels 0 to 2, a DMS should thus be used continuously. At Levels 1 and 2, for monitoring the state of the driver, a vehicle-related indicator of driving performance should be either avoided or used only when compatible with the DS features that are engaged. The speed cannot, for instance, be used as an indicator of the driver state when an adaptive cruise control is regulating this speed. As more and more DS features are introduced in vehicles, vehicle-related indicators of driving performance become less and less relevant for monitoring the state of the driver, whereas, driver-related parameters (both physiological and behavioral) remain reliable indicators.

At any of Levels 3 to 5, and when the corresponding AD features are engaged, the driver is no longer in charge of the driving task and does not need to supervise them. Additionally, at Level 3, and at any time, the driver must, however, be fallback-ready, namely, ready to take over the control of his/her vehicle when the AD features request it (that is, ask for it). A DMS should, therefore, be capable of (1) assessing whether the current state of the driver allows him/her to take over the control of his/her vehicle if requested now or in the near future, and of (2) monitoring his/her state as long as he/she is in control. El Khatib et al. [5] discuss the potential need for a DMS even when the vehicle is in control and does not require the driver to supervise the driving or to monitor the driving environment. Whenever the driver has the option of, for example, engaging in some entertainment activity, he/she must be prepared to regain control in due course. Therefore, at Level 3, despite that the driver is allowed to perform a secondary task, a DMS is still necessary to ensure that the driver is ready to take control at any time. Although the findings of various studies are sometimes contradictory, Johns et al. [6] suggest that it may be beneficial for the driver to maintain a certain level of mental workload while his/her vehicle is operated by a DAS, as this could lead to better performance during a transfer of control from automated to manual.

At Level 4, the AD features can only drive the vehicle under limited conditions, but they will not require the driver to respond within some specified time delay to a take-over request. The operational design domain (ODD) specifies the conditions under which the DAS is specifically designed to operate, including, but not limited to, (1) environmental, geographical, and time-of-day restrictions, and/or (2) the requisite presence or absence of certain traffic or roadway characteristics. Still at Level 4, the AD features are capable of automatically (1) performing a fallback of the driving task and (2) reaching a minimal-risk condition (e.g., parking the car) if the driver neither intervenes nor takes over the driving task within the delay. If the driver decides to respond to the take-over request, one can assume that the DMS would check that his/her state allows for this, even though the SAE J3016 does not say so explicitly.

At Level 5, the driving is fully automated under all possible conditions, and no DMS is required as the driver is never in control, and becomes, in effect, a passenger of the vehicle.

## 3. Survey of Literature on Driver Monitoring

This section describes our survey of the literature on DM and DMSs. The subsections below successively describe (1) our strategy for building an initial set of references, (2) some conclusions drawn from these references, (3) the design of a table for organizing them, (4) comments about the content of this table, and (5), (6) trends observable in it or in some references. The analysis performed here guides the developments in subsequent sections.

### 3.1. Strategy for Building Initial Set of References, and Number of These

**Figure 1:**
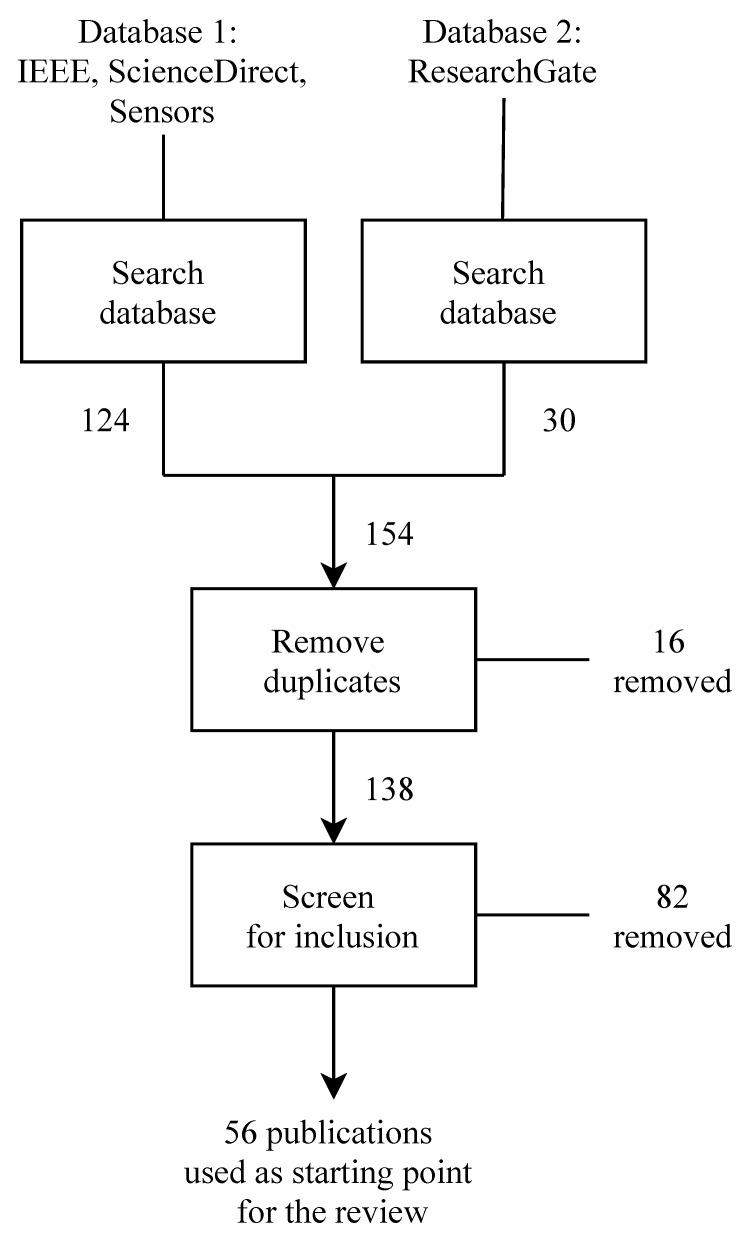
The flow diagram (1) illustrates the strategy used for our survey of the literature on driver monitoring (DM) and driver-monitoring systems (DMSs), and (2) shows the number of publications at each stage of the process.

To build an initial set of relevant references, we used an approach inspired from Gutiérrez et al. [7]. The block (or flow) diagram of Figure 1 describes it.

Our search focused on surveys, reviews, and similar studies about DM and DMSs. We independently performed two searches during February 2021. The first focused on publications from IEEE, ScienceDirect, and Sensors, and the second on publications from ResearchGate; these four databases appeared well-suited for providing a useful set of initial references. We used the search engine specific to each database and a boolean query equivalent to *(“survey” OR “review”) AND (“driver” OR “driving”) AND (“detection” OR “detecting” OR “behavior” OR “state” OR “monitoring”)*. We limited the search to publications in English, and did not place any constraint on the dates of publication. The two searches yielded 124 and 30 items, respectively. After removing 16 duplicates, we obtained a set of 138 references. We manually screened these, and only kept the ones satisfying the two criteria of (1) being in scientific journals or conference proceedings, and (2) providing a survey, review, or similar study of one or more aspects of the domain of interest. This screening led to 56 references, which appear in the first column of Table 2 and in the References section, the latter containing additional references quoted later. Appendix A provides a version of this table that is suitable for printing.

### 3.2. Conclusions from Preliminary Analysis of 56 Initial References

The preliminary analysis of the 56 initial references led to the following high-level conclusions:

1. To characterize the (global) state of a driver, one should consider the five main substates of drowsiness, mental workload, distraction, emotions, and under the influence.
2. A wide variety of parameters, which we call “indicators”, are used to characterize each of these substates, and some indicators are applicable to more than one substate.
3. Ideally, a DMS should monitor not only the driver, but also the (driven) vehicle and the (driving) environment.
4. A value for each indicator is obtained by processing data (mainly signals and images) obtained from sensors “observing” the driver, the vehicle, and the environment.
5. A DMS generally involves one or more types and/or instances of each of the following: substate, indicator, and sensor.

#### These conclusions guided the structuring and writing of the bulk of the paper

When the context is clear, we use “state” for the global state and each of the five substates. The phrase “state i” and the plural “states” imply that one is talking about one substate and several substates, respectively.

### 3.3. Design of Structure of Table Organizing Initial References

We used the above conclusions to design the structure of a table—namely Table 2—for organizing the 56 initial references in a useful way, in particular for the later synthesis in this paper.

The 56 references are listed in the first column, labelled “References”, by alphabetical order of first author. The three megacolumns following the first column successively correspond to the three key items above, and are accordingly labelled “States”, “Indicators”, and “Sensors”. The last column, labelled “Tests”, indicates whether the technique or system described in a reference was tested in the laboratory, or in real conditions (“in the wild”), or both.

The “States” megacolumn is divided into 5 columns corresponding to the 5 (sub)states of interest. Each of the “Indicators” and “Sensors” megacolumns is divided into 3 columns corresponding to the 3 previously-listed items that a DMS should ideally monitor, that is, the driver, vehicle, and environment. The column corresponding to the indicators for the driver is further divided into 3 subcolumns corresponding to the qualifiers “physiological”, “behavioral”, and “subjective”. Some other columns could be further subdivided, such as for “Distraction”, but the table deals with such additional subdivisions in a different way.

### 3.4. Description of Content of Table of References

We successively describe the three megacolumns of Table 2.

#### 3.4.1. States

For each of the 56 papers, we indicate which particular (sub)state(s) it addresses. If a paper addresses drowsiness, we place the checkmark “V” in the corresponding column, and similarly for mental workload. For the three other states, we either use a general “V” or give more specific information, often via an abbreviation. There are four types of distraction, that is, manual, visual, auditory, and cognitive, respectively abbreviated via man, vis, aud, and cog. These types are self-explanatory, but they are addressed later. For emotions, we indicate the type, that is, stress or anger (ang). For under the influence, we also indicate the type; in all cases, it turns out to be alcohol (alc).

As an example, the second paper, by Alluhaibi et al. [9], addresses drowsiness, distraction, and the emotion of anger.

All abbreviations used in Table 2, for this and other (mega)columns, are defined in Table 3.

#### 3.4.2. Indicators

The indicator(s) used by a paper is (are) indicated in the same way as above.

#### 3.4.3. Sensors

The sensor(s) used by a paper is (are) indicated in a similar, but not identical, way. If a sensor is embedded in a mobile device (typically a smartphone), rather than in the vehicle, we add a “*”, leading to “cam*”/”mic*” for a camera/microphone of a mobile device. In the vehicle column, “V” indicates that the sensor is integrated in the vehicle, whereas “V*” indicates that it is part of a mobile device. For example, the vehicle speed can be obtained via the controller-area-network (CAN) system/bus or a mobile device.

### 3.5. Trends Observable in Table

Table 2 reveals the following trends.

Drowsiness is the most covered state (with 44 references among the total of 56), distraction is the second most covered (with 20 references), and more than one (sub)state is considered in only 19 references.

Indicators are widely used in most references, in various numbers and combinations. Subjective indicators are not frequent (which is to be expected given the constraints of real-time operation). While several authors, such as Dong et al. [21] and Sahayadhas et al. [47], emphasize the importance of the environment and of its various characteristics (e.g., road type, weather conditions, and traffic density), few references (and, specifically, only 6) take them into account.

While the three “Sensors” columns seem well filled, several references either neglect to talk about the sensor(s) they use, or cover them in an incomplete way. Some references give a list of indicators, but do not say which sensor(s) to use to get access to them. References simply saying that, for example, drowsiness can be measured via a camera or an eye tracker do not help the reader. Indeed, these devices can be head- or dashboard-mounted, and they can provide access to a variety of indicators such as blink dynamics, PERCLOS, and gaze parameters.

Many systems are tested in real conditions, perhaps after initial development and validation in a simulator. Many papers do not, however, document systematically the test conditions for each method that they describe.

### 3.6. Other Trends Observable in References

Other trends are not directly observable in Table 2, but can be identified in some individual references.

Experts agree that there does not exist any globally-accepted definition for each of the first four states that we decided to consider. For example, even though many authors try to give a proper definition for drowsiness, there remains a lot of confusion and inconsistencies about the concepts of drowsiness and fatigue, and the difference between them. There is thus a need to define, as precisely as possible, what the first four states are, and this is done in the sequel.

In the more recent references, one sees a trend, growing with time, in the use of mobile devices, and in particular of smartphones [5,9,14,15,24,25,34,37,40,55]. A smartphone is relatively low-cost, and one can easily link it to a DMS. This DMS can then use the data provided by the smartphone’s many sensors, such as its inertial devices, microphones, cameras, and navigation system(s). A smartphone can also receive data from wearable sensors (e.g., from a smartwatch), which can provide information such as heart rate (HR), skin temperature, and electrodermal activity (EDA). A smartphone can also be used for its processing unit.

## 4. Driver-State Characterization via Triad of States, Indicators, and Sensors

Our survey of the field of DM and DMSs led us to the idea of synthesizing this field in terms of the three key components of states, indicators, and sensors. The next two subsections discuss the first two components, and the third subsection brings all three components into a system block diagram (BD).

### 4.1. States

Our survey convinced us that the (global) state of a driver should be characterized along at least the five dimensions—called here states—of drowsiness, mental workload, distraction, emotions, and under the influence.

One goal of a DMS is to determine the levels of one or more of these states in real time, nearly continuously, and, preferably, in a non-invasive way. We use “level” in a very general sense. The level can take several forms, such as a numerical value or a label. The numerical value can be on a continuous scale or on a discrete scale. A label can be the most likely (output) class of a classifier together with its probability, likelihood, or equivalent. A level can be binary, e.g., 0 and 1, or “alert” and “drowsy”. The levels of one or more of the five states can then be used to issue alerts or take safety actions; this is, however, not the object of this paper.

The first four states present a formidable challenge in that they are not defined in a precise way and cannot be measured directly, by contrast with, say, physical quantities such as voltage and power. The fifth state can be defined precisely, at least in the case of alcohol, but the measurement of its level requires asking the driver to blow in a breathalyzer and/or to submit to a blood test, both of which can be performed neither in real time nor non-invasively. In short, for all practical purposes, one cannot directly measure or obtain the level of any of the five states in any simple way. This is the reason for having recourse to “indicators” of each of these states.

### 4.2. Indicators

While one may have an intuitive idea of what an indicator is, it is useful to define, as precisely as possible, what it is. In a nutshell, an indicator must be well defined, and there must be a clear procedure for computing its values (at a succession of time instants) based on input data provided by one or more sensors.

For the purpose of this paper, a “quantity” or “item” is called an indicator for a given (sub)state if it satisfies all of the following conditions:

- it has a precise definition based on science (e.g., physics, mechanics, chemistry, biology, physiology);
- it can be measured, or characterized in some way, with real-time constraint when necessary, based upon data obtained from relevant sensors available in the application of interest;
- it must take values (such as numbers or labels) within a pre-specified domain, and these values must preferably correspond to physical units (such as seconds or Hertz);
- it is not a unique and full descriptor of the state;
- it is recognized, in the literature, as being linked, in some meaningful way, to the state or trend thereof;
- it is possibly useful with respect to one or more related, or unrelated, states;
- it is reproducible, meaning that its value is always the same for fixed data.

For example, the eye-blink rate (that is, the blink rate of the left or right pair of eyelids) is scientifically recognized as being indicative of drowsiness. This parameter obeys all conditions above, and is thus an indicator of drowsiness.

Similarly to the level of a state, we talk about the value of an indicator. We use both “value” and “level” simply as a way to implicitly communicate wether one is talking about an indicator or a state. Ultimately, a set of values of the indicators of a state must be converted into a level of this state. The conversion may require the use of an advanced, validated algorithm.

Indicators are generally imperfect. In most cases, an indicator cannot be guaranteed to be fully correlated with a related state. Due to the presence of complex interrelationships between each (sub)state and its indicators, it is important to use as many indicators as possible to promote a valid and reliable interpretation of the (sub)state of the driver and, ultimately, of the (global) state of the driver. An example follows. The heart rate (HR) is known to be an indicator of drowsiness. But, imagine that one relies solely on the HR to monitor drowsiness, and that the driver must suddenly brake to avoid an accident. Inevitably, this will cause his/her HR to undergo important variations. These particular variations have, however, no direct link with his/her level of drowsiness. Thus, while it is true that the HR is an indicator of drowsiness, one cannot rely on it alone to provide a reliable level of drowsiness. The environment, among other things, needs to be considered.

The values of indicators are obtained through algorithms applied to data collected via sensors.

### 4.3. System View of Characterization of a (Sub)State

**Figure 2:**
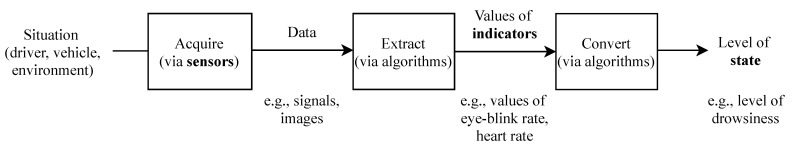
The figure shows, for the context of driver monitoring (DM), the system block diagram applicable to the characterization of a generic (sub)state. The input is the situation of interest and the output is the level of the state. The operation of each of the three subsystems is described in the text.

Figure 2 shows a system BD that uses the terminology introduced above, that is, sensors, indicators (and values thereof), and states (and levels thereof). The BD is drawn for a single, generic state, and one must specialize it for each of the five states of interest (or others).

The BD is self-explanatory. The input is the situation of interest (with the driver, vehicle, and environment). One or more sensors acquire data, typically signals and images. Algorithms extract the values of the indicators that are deemed relevant for the state of interest. Other algorithms convert these values into a level of the state. The three successive subsystems are labelled with the operation they perform, that is, acquire, extract, and convert. The input and output of each subsystem should ideally be viewed as being functions of time.

If several states are used simultaneously, the value of a given indicator can be used to compute the level of any state that this indicator relates to.

## 5. Synthesis of Driver-State Characterization via Two Interlocked Tables

The previous section shows the key role played by the triad of states, indicators, and sensors (also emphasized in Figure 2) in driver-state characterization, which is the first of two key steps in DM, and the object of this paper. The present section describes our approach to synthesize, in terms of this triad, the techniques for driver-state characterization found in the literature.

**Figure 3:**
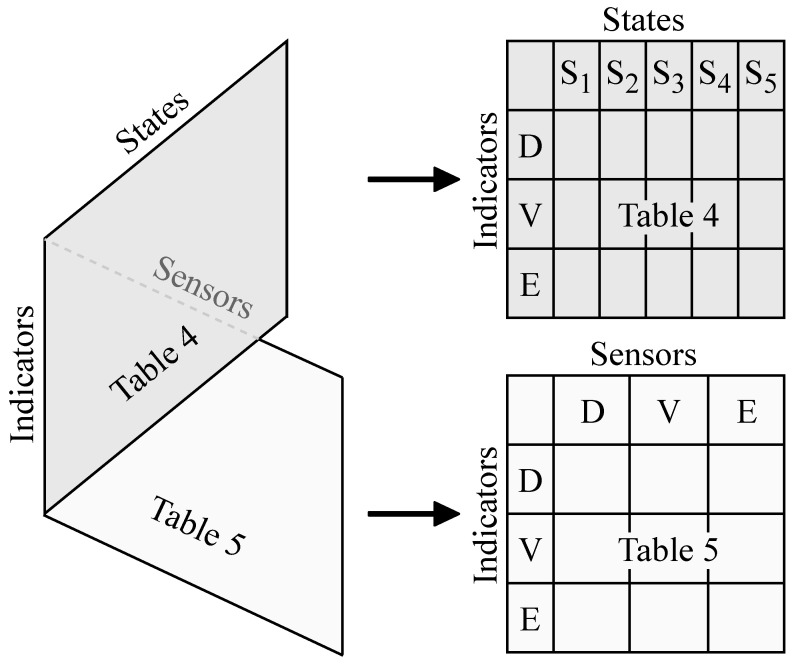
The figure shows simplified representations of key Table 4 (states vs. indicators) and Table 5 (sensors vs. indicators). It also suggests that these tables can naturally be interpreted as being two views of an underlying 3D array. $S_{i}$, D, V, and E stand for “State i”, “Driver”, “Vehicle”, and “Environment”, respectively.

Our approach aims at answering, in a simple, visual way, the two following questions: (1) For a given state, what indicator(s) can one use? (2) For a given indicator, what sensor(s) can one use? We achieve this goal by naturally providing two tables (or matrices) of “states vs indicators” and “sensors vs indicators”. These two tables can be viewed as being two-dimensional (2D) views of a 3D table (or array) of “states vs indicators vs sensors”, as illustrated in Figure 3, where the positions shown for the three dimensions and for the “dihedral” they subtend make the tables on the right appear in numerical order from top to bottom. The figure shows visually that the tables share the “Indicators” dimension, and are thereby interlocked. It gives a simplified representation of each of the tables that are progressively filled in Section 6, Section 7, Section 8, Section 9 and Section 10, that is, Table 4 and Table 5.

### 5.1. Preview of Two Key Tables

In Figure 3, the simplified representations of Table 4 and Table 5 give the high-level structures of these tables.

In Table 2, the megacolumn “Indicators” is partitioned into the three columns “Driver”, “Vehicle”, and “ Environment”. Figure 3 shows, via the simplified representations, that Table 4 and Table 5 are also partitioned in this way, but in megarows and with the corresponding abbreviations D, V, and E. In Table 2, the megacolumn “Sensors” is partitioned in the same way as the megacolum “Indicators”. This is reflected in Figure 3 by the partitioning of Table 5 into the megacolumns D, V, and E. The figure shows that Table 4 is partitioned into the five megacolumns corresponding to the five states, denoted here by $S_{1},\;\ldots ,\;S_{5}$, where $S_{i}$ stands for “State i”. This quoted phrase appears at the beginning of the titles of the next five sections, with the successive values of i.

Each lowest-level cell in both tables is destined to contain 0, 1, or more related references.

The pair of tables allows one to answer other questions such as: (1) If one invests in the calculation of an indicator for a particular state, what other state(s) can this indicator be useful for? (2) If one invests in a particular sensor for a particular state, what other state(s) can this sensor be useful for?

### 5.2. Further Subdivision of Rows and Columns

The rows and columns of Table 4 and Table 5 are further divided as follows. The D-megarows of Table 4 and Table 5 are subdivided as the D-megacolumns of Table 2 are, that is, into the rows “Physiological”, “Behavioral”, and “Subjective”.

The D-megacolumns of Table 5 are subdivided in a way that does not already appear in Table 2, that is, into the columns “Seat”, “Steering Wheel”, “Safety Belt”, “Internal Camera”, “Internal Microphone”, and “Wearable”. Observe that the D-megarows and D-megacolumns are not subdivided in the same way, even though they correspond to the driver.

The V- and E- rows and columns are also further divided as necessary.

### 5.3. Categories of Indicators and Sensors

We give examples of the various categories of indicators and sensors that are further discussed in the next five sections. Below, we use the self-explanatory terminology of "X-based indicators" and "X-centric sensors", where X can be replaced by driver (or D), vehicle (or V), or environment (or E).

#### 5.3.1. Indicators

D-based indicators relate to the driver. They include physiological indicators (e.g., heart activity, brain activity, electrodermal activity (EDA)), behavioral indicators (e.g., eye blinks, gaze direction, hands positions), and subjective indicators (which are not suited for real-world operation, but can be used for validation at some point in the development of a DMS).

V-based indicators relate to how the driver controls his/her vehicle, for example, how he/she controls the speed, steers, and brakes.

E-based indicators relate to the environment, viewed here as consisting of three parts, that is, (1) the outside environment (outside of vehicle), (2) the inside environment (inside of vehicle), and (3) the contextual environment (independent of the previous two). Examples of characteristics of these parts of the environment are, respectively, (1) the road type, weather conditions, and traffic density; (2) the temperature and noise; and (3) the time of day and day of year. Each of these characteristics (e.g., road type) can be used as an E-based indicator.

#### 5.3.2. Sensors

Some D-centric sensors are placed in the seat (e.g., radar for breathing activity), steering wheel (e.g., electrodes for electrocardiogram (ECG)), and safety belt (e.g., magnetic induction (MI) sensors). Some D-centric sensors, in particular cameras (e.g., RGB) and microphones, are appropriately placed in the cockpit to monitor the driver. We qualify these sensors of “internal”, to distinguish them from similar sensors monitoring the external environment, and qualified of “external”. Some D-centric sensors are wearables (e.g., a smartwatch measuring HR and/or skin temperature). Since the aim is to monitor the state of the driver, we assume throughout this paper that the seat, safety belt, and similar items are related to the driver.

V-centric sensors are mostly sensors—whether integrated in the vehicle or not—that allow for the acquisition of vehicle parameters such as speed, steering angle, and braking level. Such parameters are often obtained via the CAN bus. Sensors (e.g., accelerometers, gyroscopes) built into recent mobile devices can, however, also provide some of this information.

E-centric sensors are sensors that allow for the acquisition of parameters related to the environment. Cameras and radars can provide, for example, information about the driving scene.

### 5.4. Preview of Next Five Sections

The next five sections successively cover the five selected states in detail. In general, each section defines a state, the indicators that characterize it, and the sensors that allow access to them, and progressively fills Table 4 and Table 5 with relevant references.

At the end of the last of these five sections, both tables are complete. They, together with the explanations in the five sections, constitute the main contribution of this paper.

The structures of Table 2, Table 4, and Table 5 were obtained after a significant number of iterations. This implies that the ultimate structure of Table 2 was informed by the content of Section 4, Section 5, Section 6, Section 7, Section 8, Section 9 and Section 10.

## 6. State 1: Drowsiness

We provide a detailed description of (the state of) “drowsiness”, and we then present the indicators and sensors that can be used to characterize it.

### 6.1. Description

Johns [63] appears to have provided the earliest, accurate definition of drowsiness, that is, the state of being drowsy. Massoz [64] provides useful, recent information about this state. Drowsiness is an intermediate arousal state between wakefulness and sleep, that is, between being awake and being asleep; it thus refers to a state just before potential sleep. A drowsy person has both a difficulty to stay awake and a strong inclination to sleep. It is a continuous, fluctuating state of (1) reduced awareness of the “here and now” [65] and (2) impaired cognitive and/or psychomotor performance. It is often the result of a monotonous activity, such as a long drive on a monotonous road. It can have a detrimental effect on the safety of driving. For example, in the USA in 2018, there were 785 fatal accidents due to drowsiness for a total of 36,835 people killed in motor vehicle crashes and, in 2019, these numbers were 697 vs. 36,096 [66]. It can be viewed as a state of basic physiological need like hunger and thirst, that is, as an indication that one needs to sleep. It can be considered to be synonymous with sleepiness, somnolence, and sleepening, the latter being a less common term meaning “entry into sleep” [67].

Drowsiness is, however, not synonymous with fatigue. These are two distinct physiological states that are often confused, even in the scientific literature. Fatigue corresponds to the feeling of being tired or exhausted as a result of long periods of physical activity and/or cognitive activity. It is characterized by an increasing difficulty to accomplish an effort linked to a task. It can be considered to be synonymous with tiredness. Talking about fatigue helps one to further narrow down what drowsiness is and is not.

May and Baldwin [68] suggest that, for driving, one should distinguish between sleep-related (SR) fatigue and task-related (TR) fatigue, based on the causing factors. SR fatigue can be caused by sleep deprivation, long wakefulness, and time of day (with effect of circadian rhythm), while TR fatigue can be caused by certain characteristics of driving, like task demand and duration, even in the absence of SR fatigue. These suggested subcategories of fatigue clearly intersect with drowsiness, but it is difficult to say exactly how.

Fatigue can be alleviated by taking a break (without necessarily sleeping), while drowsiness can be alleviated by sleeping, even by taking a nap or a power nap. One can be drowsy without being fatigued and vice-versa, and one can be both. Fatigue and drowsiness both lead to decrements in performance. In practice, it is difficult to distinguish between them, and even more to quantify how much of a decrement is due to each of them individually, especially in real time and non-invasively. Their indicators appear to be mostly the same. In the driving context, one focuses on monitoring drowsiness, with the main goal of preventing the driver from falling asleep at the wheel.

There are many publications about the various ways of characterizing drowsiness [64,69,70,71], and apparently fewer for fatigue [72]. Very few papers tackle both phenomena [73].

### 6.2. Indicators

We start with the driver-based indicators, divided into the three categories of physiological, behavioral, and subjective indicators.

The most substantial changes in physiology associated with changes in the level of drowsiness (LoD) lie in the brain activity as measured by the electroencephalogram (EEG). Tantisatirapong et al. [74] model EEG signals using the fractal Brownian motion (fBm) random process. They carried out experiments in a driving simulator, and considered the three time periods of before, during, and after sleep, where they mimic sleep by asking the driver to close his/her eyes, pretending to try to fall asleep. They saw corresponding changes in the computed fractal dimension (related, for self-replicating random processes, to the Hurst exponent), which allows them to classify the driver as alert or drowsy. They conclude that the fractal dimension of an EEG signal is a promising indicator of drowsiness. Changes in physiology also manifest themselves in the heart activity, as measured by the ECG. Indeed, as drowsiness increases, the HR decreases and the heart rate variability (HRV) increases [75]. However, HRV data vary both between individuals and over time for each individual, depending on both internal and external factors. Therefore, the many confounding factors that also influence HRV must be accounted for in order to use HRV as an indicator of drowsiness [76]. The breathing activity is an indicator of drowsiness, as changes in breathing rate or inspiration-to-expiration ratio occur during the transition from wakefulness to drowsiness [77]. Drowsiness leads to changes in EDA, also called skin conductance or galvanic skin response (GSR), which relates to the electrical resistance measured via electrodes placed on the surface of the skin. The skin resistance fluctuates with sweating, the level of which is controlled by the sympathetic nervous system, which autonomously regulates emotional states such as drowsiness [78]. The pupil diameter instability has been linked to drowsiness. Indeed, several studies found that the pupil diameter fluctuates at a low frequency and with a high amplitude whenever a subject reports being drowsy [79,80,81].

Eye behavior is a good indicator of drowsiness. In a clinical setting, one traditionally characterizes this behavior by electrooculography (EOG) [82], which implies the use of electrodes. In operational settings where a non-invasive characterization is highly desirable, one generally uses video sequences of the eye(s) and applies image-analysis methods to them. The dynamics of eye closures (in particular, long and slow closures) is recognized as a strong and reliable indicator of drowsiness [83]. The most-standard indicator of spontaneous eye closure is the percentage of closure (PERCLOS) [84,85,86]. It is usually defined as the proportion of time (over a given time window) that the eyelids cover at least 70% (or 80%) of the pupils. As the LoD increases, the eye closures become slower and longer, and the upper eyelid droops, and all of this contributes to an increase in PERCLOS. Other reliable, standard indicators include mean blink duration [83,87], mean blink frequency or interval [83,88], and eye closing and reopening speeds [83]. Recently, Hultman et al. [89] used electrophysiological data obtained by EOG and EEG to detect drowsiness with deep neural networks, and found that, for driver-drowsiness classification, EOG data (and, more precisely, the related blink data) are more informative than EEG data.

All the above elements constitute objective indicators of drowsiness. Besides these, there are subjective indicators, consisting of questionnaires and self-reports. While they are not suitable for real-time characterization of drowsiness, they can be used to validate other indicators, as ground truth to train models, and/or to evaluate the performances of systems. These subjective indicators include the Karolinska sleepiness scale (KSS) [90], the Stanford sleepiness scale (SSS) [91], and the visual analog scale (VAS) [92].

The above information allows one to fill the cells of Table 4 at the intersection of the “Drowsiness” column and the “Driver” megarow. The latter lists a total of fourteen indicators. We stress that these may or may not be relevant for each of the five states.

A cell (at the lowest level) in the heart of Table 4 is either empty or filled with one or more related reference(s). For example, this table shows that we found three significant references about “pupil diameter” as an indicator of drowsiness, that is, [79,80,81], while we found no significant reference about “gaze parameters” as an indicator of drowsiness. The table shows, however, that we found references reporting that this last indicator is useful for the state of emotions (discussed later).

Below, as we progressively fill Table 4 and Table 5, we simply indicate which cell(s) is/are concerned. As we progress, the discussion in the last two paragraphs remains valid, after proper adaptation.

As should be clear from this discussion, the finer hierarchical partitioning of Table 4 and Table 5 into the lowest-level columns and rows is progressively obtained from the developments in Section 3, Section 4, Section 5, Section 6, Section 7, Section 8, Section 9 and Section 10.

We now consider the vehicle-based indicators. In the literature, they are often called measures of driving performance, the latter being known to degrade with increasing drowsiness [93,94,95]. These indicators characterize the driving behavior. Common such indicators include speed, lateral control (or lane discipline), braking behavior, and wheel steering. These last indicators are found in the central part of Table 4, next to the “Vehicle” header.

The main vehicle-based indicator of drowsiness is the standard deviation of lane position (SDLP) [96,97,98,99]. As the term suggests, SDLP measures the driver’s ability to stay centered in his/her lane. Drowsiness can also produce greater variability in driving speed [100]. Another important vehicle-based indicator is the steering wheel movement (SWM) [97]. It has been shown that a drowsy driver makes fewer small SWMs and more large ones. When a driver loses concentration, the vehicle begins to drift away from the center of the lane, but, when the driver notices the drift, he/she compensates by large SWMs toward the lane center [101].

Jacobé de Naurois et al. [102] conducted a study in a driving simulator, using different artificial neural networks (ANNs) based on various data, to detect drowsiness and predict when a driver will reach a given LoD. The data used are either (1) driver-based, physiological indicators (HR, breathing rate) and behavioral indicators (blinks, PERCLOS, head pose), or (2) vehicle-based indicators (lane deviation, steering wheel angle, acceleration, speed). The results of the study show that the best performance is obtained with behavioral data, successively followed by physiological data and vehicle data, for both detection and prediction.

Most real-time, drowsiness-monitoring systems characterize the LoD at the “present” time using sensor data located in a sliding time window butting against this present time. Therefore, this LoD corresponds, not to the present, but to roughly the center of the window, thus several seconds, or tens of seconds, in the past. If this “present” LoD is above a dangerous level, it may be too late for the driver or the vehicle to take proper action. Given that, at 100km/h, it takes about 2 s to get out of lane (then possibly hitting an obstacle), predictions just 10 to 20 s into the future would already help. It is thus crucial to be able to predict (1) the future evolution of the LoD and (2) the associated risks.

Ebrahimbabaie [69] and Ebrahimbabaie and Verly [103] developed and tested a prediction system that (1) takes as input a discrete-time, validated LoD signal consisting of the past LoD values produced at regular intervals, up to just before the present time, as in [70,104] (discussed later), and (2) produces as output several types of predictions. Treating the LoD signal as a realization of an underlying random process (RP), the authors investigate the use of the RPs called “autoregressive (integrated) moving average (AR(I)MA)” (from time-series analysis) and “geometric Brownian motion (GBM)” (found almost exclusively in finance). They show that the LoD signal can generally be modeled as AR(I)MA and GBM within each position of the sliding window (thus locally), they estimate the parameters of the model for each position of the window, and they use them to make predictions of one or more of the following three types: future values of LoD signal, first hitting time (of a critical LoD threshold), and survival probability.

We emphasize that “to predict” means “to tell beforehand”, and thus, in the present context, to use past data to compute now a quantity that describes some future situation. In the literature, this “future situation” often turns out to be a “present situation”, so that no prediction is performed.

The above information allows one to fill, in Table 4, the relevant cells of the “Drowsiness” column and the “Vehicle” megarow.

Note that there are no entries in the “Environment” megarow of the “Drowsiness” column, which means that we did not find any significant technique that uses one or more indicators related to one of the three parts of the environment listed in Section 5.3 (that is, outside, inside, and contextual) to determine the level of drowsiness of the driver. Some papers attempt to use the time of day to try to capture the moments of the day where drowsiness tends to peak. While the monotonicity of a road is known to increase driver drowsiness, we have not found any paper using environment-based indicators of road monotonicity (e.g., road geometry or traffic density), and describing a way to give values to such indicators based upon available data. As an aside, studies of drowsiness in a driving simulator often use night driving and monotonous conditions to place the driver in a situation conducive to drowsiness.

### 6.3. Sensors

Similarly to the indicators, we first address the driver-centric sensors.

In a vehicle, the HR can be monitored using electrodes that can be placed at various locations, including the steering wheel (conductive electrodes [105]) and the seat (capacitive electrodes [106]). ECG monitoring using steering-wheel-based approaches is a feasible option for HR tracking, but requires both hands to touch two different conductive parts of the steering wheel.

Ballistocardiography (BCG) also allows for monitoring the cardiac activity unobtrusively. The underlying sensing concept uses strain-gauge BCG sensors in the seat or in the safety belt to detect both the cardiac activity and the respiratory activity of the driver [107]. However, the vehicle vibrations make it difficult to use this sensor in real driving conditions.

Information about the cardiac activity can be obtained using a camera looking at the driver, in particular using photoplethysmography (PPG) imaging [108].

Radar-based methods mainly provide information about movement, which can of course be caused by both the cardiac activity and the respiratory activity. Various sensor locations are possible, including integration into the safety belt, the steering wheel, and the backrest of the seat [109,110].

Thermal imaging is a tool for analyzing respiration (or breathing) non-intrusively. Kiashari et al. [77] present a method for the evaluation of driver drowsiness based on thermal imaging of the face. Indeed, temperature changes in the region below the nose and nostrils, caused by inspiration and expiration, can be detected by this imaging modality. The procedure (1) uses a sequence of infrared (IR) images to produce a corresponding discrete-time signal of respiration, and (2) extracts respiration information from it. (Unless indicated otherwise, infrared (IR) means long-wave IR (LWIR), that is, with wavelengths of 8–14μm; LWIR is the “thermal” range of IR.) The value of each successive signal sample is the mean of the pixels in a rectangular window of fixed size, representing the respiration region in the corresponding IR image, adjusted frame-to-frame using a tracker. The initial respiration region is determined based on the temporal variations of the first few seconds of the sequence, and the region is tracked from frame-to-frame by using the technique of “spatio-temporal context learning” [111], which is based on a Bayesian framework, and models the statistical correlation between (1) the target (that is, the tracked region) and (2) its surrounding regions, based on the low-level characteristics of the image (that is, the intensity and position of each pixel). The extracted information is the respiration rate and the inspiration-to-expiration ratio. A classifier uses these rate and ratio to classify the driver as awake or drowsy. A support vector machine (SVM) classifier and a *k*-nearest neighbors (KNN) classifier are used, and the first does result in the best performance.

François [70] and François et al. [104] describe a photooculographic (POG) system that illuminates one eye with eye-safe IR light and uses as input a sequence of images of this eye acquired by a monochrome camera that is also sensitive in this IR range, and is head-mounted or dashboard-mounted. A large number of ocular parameters, linked to the movements of the eyelids (including blinks) and eyeball (including saccades), are extracted from each video frame and combined into an LoD value, thus producing an LoD signal. The output was validated using EEG, EOG, EMG, and reaction times. The head-mounted system is available commercially as the Drowsimeter R100.

Using a camera, Massoz et al. [112] characterize drowsiness by using a multi-timescale system that is both accurate and responsive. The system extracts, via convolutional neural networks (CNNs), features related to eye-closure dynamics at four timescales, that is, using four time windows of four different lengths. Accuracy is achieved at the longest timescales, whereas responsiveness is achieved at the shortest ones. The system produces, from any 1-min sequence of face images, four binary LoDs with diverse trades-offs between accuracy and responsiveness. Massoz et al. [112] also investigate the combination of these four LoDs into a single LoD, which is more convenient for operational use.

Zin et al. [113] classify driver drowsiness by using a feature-extraction method, the PERCLOS parameter, and an SVM classifier.

EDA is measured through electrodes placed on the skin of a person. It can thus be measured through a wearable such as a smartwatch. Concerning the other, relevant, physiological, driver-based indicators, (1) it is challenging to get the pupil diameter in real conditions because of issues with illumination conditions and camera resolution, among others reasons, and (2) it is nearly impossible, as of this writing, to characterize brain activity in real time and in a non-intrusive, reliable way.

Teyeb et al. [114] measure vigilance based on a video approach calculating eye-closure duration and estimating head posture. Teyeb et al. [115] monitor drowsiness by analyzing, via pressure sensors installed in the driver seat, the changes in pressure distribution resulting from the driver’s body moving about in this seat. The authors suggest that the techniques of these two papers can be usefully combined into a multi-parameter system.

Bergasa et al. [116] present a system to characterize drowsiness in real time using images of the driver and extracting from them the six visual parameters of PERCLOS, eye-closure duration, blink frequency, nodding frequency, fixed gaze, and face pose. Using a camera, Baccour et al. [117] and Dreiβig et al. [118] monitor driver drowsiness based on eye blinks and head movements.

Vehicle-based indicators can be collected in two main ways. Standard indicators such as speed, acceleration, and steering wheel angle, can be extracted from CAN-bus data [119,120]. The CAN bus enables intra-vehicle communications, linking the vehicle sensors, warning lights, and electronic control units (ECUs). More advanced indicators can be obtained in appropriately-equipped vehicles [119,121]. For example, speed and acceleration can be obtained via an inertial measurement unit (IMU), and following distance via a forward-looking radar.

Since SDLP is considered to be a vehicle-based indicator of driver drowsiness, one can quantify this indicator by examining the lane discipline, that is, the behavior of the vehicle in its lane. This is traditionally done by using cameras (mounted inside, behind the windshield, typically integrated beside the rear-view mirror) [122] and/or laser sensors (mounted at the front of the vehicle) to track the lane-delimiting lines when present. However, one can also use the rumble strips (also called sleeper lines, audible lines, or alert strips) when present. While these are designed to produce an audible, acoustic signal intended to be sensed directly by the driver (as an urgent warning or wake-up call), one could imagine using microphones and/or vibration sensors to transform this acoustic/mechanical signal into an electrical signal that is then analyzed via signal processing.

Bakker et al. [123] describe a video-based system for detecting drowsiness in real time. It uses computer vision and machine learning (ML), and was developed and evaluated using naturalistic-driving data. It has two stages. The first extracts, using data from the last 5 min (1) driver-based indicators (e.g., blink duration, PERCLOS, gaze direction, head pose, facial expressions) using an IR camera looking at the driver’s face, and (2) vehicle-based indicators (e.g., lane positions, lane departures, lane changes) using an IR camera looking at the scene ahead. This stage mostly uses pre-trained, deep-neural-network (DNN) models. All indicators—also called deep features in DNNs—are inputs to the second stage, which outputs an LoD, either binary (alert or drowsy) or regression-like. This stage uses one KNN classifier, trained and validated using KSS ratings as ground truth for the LoD, and personalized for each driver by weighting more his/her data during training, thereby leading to higher performance during operation.

The above information allows one to fill the relevant cells of Table 5.

## 7. State 2: Mental Workload

We provide a detailed description of (the state of) “mental workload”, and we then present the indicators and sensors that can be used to characterize it.

### 7.1. Description

Mental workload, also known as cognitive (work)load (or simply as driver workload in the driving context), is one of the most important variables in psychology, ergonomics, and human factors for understanding performance. This psychological state is, however, challenging to monitor continuously [124]. In this section, we consider “mental” and “cognitive” to be synonyms.

A commonly-used definition of mental workload is the one proposed by Hart and Staveland [125]. They define mental workload as the cost incurred by a person to achieve a particular level of performance in the execution of a task. It is thus the portion of an individual’s mental capacity—necessarily limited—that is required by the demands of this task [126,127], that is, the ratio between the resources required to perform it and the available resources of the person doing it [128,129].

In the literature on mental workload, one often finds references to another state called cognitive distraction. Mental workload and cognitive distraction are two different concepts, even if they can be linked when a driver performs secondary tasks while driving. Cognitive distraction increases the mental workload of a driver. An increase in mental workload is, however, not in itself an indication of cognitive distraction. First, mental workload can increase in the absence of distraction, for example, when a driver is focusing to execute the primary task of driving correctly and safely. Second, mental workload can increase significantly with an increasing complexity of the driving environment [130]. Cognitive distraction is further considered later as a particular category of (the state of) distraction.

Mental workload and stress are also linked since an increasing mental workload usually induces some stress in the driver.

### 7.2. Indicators

In the driving context, visual tasks and mental tasks are closely linked. Indeed, while driving, a driver is constantly perceiving his/her driving environment and analyzing what he/she sees in order to make the right decisions whenever required, for example, scanning a crossroad and simultaneously judging the time and space relationships of other road users to decide when it is safe to cross an intersection. Therefore, it is logical that many researchers use eye-related parameters (e.g., blinks, fixations, and pupil diameter) to assess the mental workload of a driver [33].

Among the driver-based, physiological indicators, EDA [131], HR [132], and HRV [133] are often used as indicators of mental workload. HR increases as a task gets more difficult [134] or if other tasks are added [135]. EEG is also a valuable indicator for studying mental workload because it records the electrical activity of the brain itself, but it is complex to analyze [136]. The pupil diameter is considered to be an indicator of mental workload [132,137,138]. Indeed, Yokoyama et al. [139] indicate that the mental workload of a driver may be predicted from the slow fluctuations of the pupil diameter in daylight driving. All physiological parameters mentioned in this paragraph are, however, also influenced by other aspects of the mental and physical situation of the driver (e.g., drowsiness and TR fatigue) and by environmental situation (e.g., illumination and temperature).

Among the driver-based, behavioral indicators, Fridman et al. [140] have shown that the visual scanning by a driver decreases with an increasing mental workload. Furthermore, since the interval of time between saccades has been shown to decrease as the task complexity increases, saccades may be a valuable indicator of mental workload [141,142].

Subjective measures of mental workload exist, like the NASA task load index (NASA TLX) [125], which is a workload questionnaire for self-report, and the rating scale mental effort (RSME).

Driving performance can diminish as a result of an increase in mental workload. The vehicle-based indicators which are the most sensitive to such an increase are SDLP and SWM [130].

Palasek et al. [143] use the driving environment to estimate the attentional demand required from the driver to drive. The features extracted from the analysis of the driving environment are thus indicators of the mental workload of the driver.

The above information allows one to fill, in Table 4, the relevant cells of the “Mental Workload” column.

### 7.3. Sensors

Cameras are often used in the literature to characterize mental workload as they are particularly well suited to extract driver-based, behavioral indicators and are non-invasive.

Fridman et al. [140] describe a system for characterizing, non-invasively, via a camera facing the driver, what they call his/her cognitive load (CL). The system exploits the well-documented, experimental observation that the angular distribution of gaze direction (often characterized by the 2D pupil position) tends to become more concentrated, especially vertically, when the CL increases. Using video imagery, the system classifies the CL of the driver into one of the three CL levels (low, medium, high), as he/she engages in activities other than the primary task of driving, such as a conversation or the adjustment of the infotainment system. The system extracts, from a 90-frame, 6-second video clip, via computer vision, the face and the region of one eye of the driver. It then uses one of two methods: (1) mainly active appearance models (AAMs) for the face, eyelids, and pupil (when visible) to produce a sequence of pupil 2D positions, and (2) one hidden Markov model (HMM) for each of the three CL levels. The second method uses a single 3D CNN with three output classes corresponding to these levels. The two methods thus rely on a sequence of pupil positions and on a sequence of eye images, respectively. The output of the system is one of the three CL levels.

In order to develop this system, the authors first acquired training data in real-driving conditions while imposing on the driver a secondary task of a given CL level. This imposition of a given CL level while performing a primary task (here driving) is commonly achieved in the literature through the standard “*n*-back” task, where the three values of *n*, that is, *n* = 0, 1, and 2, are viewed as corresponding to low, medium, and high CL. For the *n*-back task, a sequence of numbers is dictated to the subject, who is asked, for each number, whether it matches the one dictated *n* positions earlier in the sequence. For example, for *n* = 2, the subject must indicate whether the current number is the same as the one he/she heard 2 steps before, all this while he/she performs the primary task, here driving.

The authors indicate (1) that the differences in cognitive loading for the three levels have been validated using, among others, physiological measurements (e.g., HR, EDA, and pupil diameter), self-report ratings, and detection-response tasks, and (2) that these levels have been found to cover the usual range of secondary tasks while driving, such as manipulating a radio or a navigation system.

It is noteworthy that the data used for building the system was acquired through real driving, during which the driver repeatedly performed *n*-back tasks, while a camera was recording his/her face and surrounding area, this by contrast with the many other developments made using a driving simulator, in highly controlled conditions, and difficult to implement in real-life conditions.

The authors indicate that, while they use the term “cognitive load”, the literature often uses synonyms like “cognitive workload”, “driver workload”, and “workload”.

Musabini and Chetitah [144] describe another system that is also based on eye-gaze dispersion. They use a camera facing the driver, produce a heatmap representing the gaze activity, and train an SVM classifier to estimate the mental workload based on the features extracted from this representation.

Le et al. [145] characterize the mental workload based on the involuntary eye movements of the driver, resulting from head vibrations due to changing road conditions. They report that, as the mental workload increases, these involuntarily eye movements become abnormal, resulting in a mismatch between the actual eye movements measured via an eye-tracking device and the predicted eye movements resulting from a "VOR + OKR" model, where VOR and OKR are the abbreviations of vestibular–ocular reflex and optokinetic response. For each driver, the VOR parameters are estimated during the first 10 s of driving in condition of normal mental workload, whereas the OKR parameter is fixed. The hypothesis of abnormal eye movements while driving under mental workload was validated using a t-test analysis. Different levels of mental workload were induced in a driving simulator using the *n*-back task.

Palasek et al. [143] use an external camera recording the driving environment to estimate the attentional demand using attentive-driving models. Indeed, the task of driving can sometimes require the processing of large amounts of visual information from the driving environment, resulting in an overload of the perceptual systems of a human being. Furthermore, traffic density is known to increase the mental workload [146], so that urban environments lead to a higher mental workload than rural and highway environments do [147], all other conditions being equal.

The above information allows one to fill the relevant cells of Table 5.

## 8. State 3: Distraction

By contrast with the two previous sections, we start with some background information (up to Section 8.1) on the state of distraction.

The globally accepted definition of driver distraction follows: it is a diversion of attention, away from activities critical for safe driving (the primary task) and toward a competing activity [148,149].

Inattention, sometimes used—mistakenly—as a synonym of distraction, is defined as a diminished attention to activities that are critical for accomplishing a primary task, but not necessarily in the presence of a competing activity [149]. Therefore, driver distraction is one particular form of driver inattention [150]. Inattention is a broader term as it can be caused, for example, by drowsiness. It indeed occurs in a wide range of situations in which the driver fails to attend to the demands of driving, such as when a desire to sleep overcomes a drowsy driver.

Driver distraction can be caused by any cognitive process such as daydreaming, mind wandering, logical and mathematical problem solving, decision making, using any kind of in-vehicle system, for example, for entertainment, navigation, communication (including a cell phone), and any other activity that may affect the driver’s attention to driving [151]. It is helpful to distinguish between four types of distractions [21,152]: (1) manual distraction (e.g., manually adjusting the volume of the radio); (2) visual distraction (e.g., looking away from the road); (3) auditory distraction (e.g., answering a ringing cell phone); and (4) cognitive distraction (e.g., being lost in thought). Several distracting activities may, however, involve more than one type of distraction (e.g., talking on the phone while driving creates at least an auditory distraction and a cognitive distraction, under the assumption that a hands-free system is used, thereby avoiding manual distraction).

When distracted, the driver looses awareness of the current driving situation. Being aware of a situation (whether for driving or for some other activity) is often called situational awareness (SA). A loss of SA while driving results in a reduction of vigilance and in an increase of the risk of accident. In driving, a major aspect of SA is the ability to scan the driving environment and to sense dangers, challenges, and opportunities, in order to maintain the ability to drive safely. As a driver moves through the environment, he/she must—to avoid getting into an accident—identify the relevant information in rapidly changing traffic conditions (e.g., distance to other vehicles, closing speed), and be prepared to react to suddenly-appearing events (e.g., braking because of an obstacle, obeying a road sign). To achieve SA, a driver must thus perceive correctly his/her driving environment [153], be attentive, and have a working memory [129]. It follows that any distraction that harms the driver’s attention may adversely impact SA [154].

Kircher and Ahlström [155] argue that existing definitions of distraction have limitations because they are difficult to operationalize, and they are either unreasonably strict and inflexible or suffering from hindsight bias, the latter meaning that one needs to know the outcome of the situation to be able (1) to tell what the driver should have paid attention to and, then, (2) to judge whether he/she was distracted or not. The authors are also concerned that distraction-detection algorithms (1) do not take into account the complexity of a situation, and (2) generally cover only eyes-off-road (EOR) and engagement in non-driving related activities (NDRA). They thus developed a theory, named MiRA (minimum required attention), that defines the attention of a driver in his/her driving environment, based on the notion of SA. Instead of trying to assess distraction directly, one does it indirectly, by first trying to assess attention. Recall that distraction is a form of inattention.

According to the MiRA theory, a driver is considered attentive at any time when he/she samples sufficient information to meet the demands of the driving environment. This means that a driver should be classified as distracted only if he/she does not fulfill the minimum attentional requirements to have sufficient SA. This occurs when the driver does not sample enough information, whether or not simultaneously performing an additional task. This theory thus acknowledges (1) that a driver has some spare capacity at his/her disposal in the less complex driving environments, and (2) that some glances toward targets other than the roadway in front of him/her may, in some situations, be needed for the driving task (like looking at, or for, a vehicle coming from each of the branches at a crossroad). This means that EOR and engagement in NDRA do not necessarily lead to driver distraction.

The MiRA theory does not conform to the traditional types of distraction (manual, visual, auditory, cognitive) as it does not prescribe what sensory channel a certain piece of information must be acquired through.

In an attempt to operationalize the MiRA theory, Ahlström et al. [156] present an algorithm for detecting driver distraction that is context dependent and uses (1) eye-tracking data registered in the same coordinate system as an accompanying model of the surrounding environment and (2) multiple buffers. Each buffer is linked to a corresponding glance target of relevance. Such targets include: windshield, left and right windows, (rear-view) mirrors, and instrument cluster. Some targets and their buffers are always present (like the roadway ahead via the windshield, and behind via the mirrors), while some other targets and their buffers appear as a function of encountered traffic-regulation indications and infrastructural features. Each buffer is periodically updated, and its update rate can vary in time according to requirements that are either “static” (e.g., the presence of a specific on-ramp that requires one to monitor the sides and mirrors) or “dynamic” (e.g., a reduced speed that lessens the need to monitor the speedometer). At each scheduled update time, a buffer is incremented if the driver looks at the corresponding target, and decremented otherwise; this is a way of quantifying the “sampling” (of the environment) performed by the driver. A buffer running empty is an indication that the driver is not sampling enough the corresponding target; he/she is then considered to be inattentive (independently of which buffer has run empty). Until declared inattentive, he/she is considered attentive.

This completes the background information on the state of distraction. We now successively consider the four types of distraction. For each of the four corresponding substates, we provide a detailed description, and we then present the indicators and sensors that can be used to characterize it.

### 8.1. State 3.1: Manual Distraction

#### 8.1.1. Description

Manual distraction, also called biomechanical distraction, occurs when the driver is taking one or both of his/her hands off the steering wheel. The driver may do so to answer a call or send a text message, grab food and eat, or grab a beverage and drink, all while driving. According to the National Highway Traffic Safety Administration (NHTSA), texting while driving is the most alarming distraction. It is mainly due to manual distraction, but, inevitably, it also includes both visual distraction and cognitive distraction.

#### 8.1.2. Indicators

Unsurprisingly, the best indicator used to detect manual distraction is the behavior of the driver’s hands, mainly through their positions and movements. For safe driving, these hands are expected to be, most of the time, exclusively on the steering wheel, the gearshift, or the turn-signal lever. On the contrary, a hand using a phone, adjusting the radio, or trying to grab something on the passenger seat indicates a manual distraction [157].

Vehicle-based indicators can also be used, as shown in [158]. Using naturalistic-driving data, the authors studied the correlation between (1) performance metrics linked to the steering-wheel behavior and to the vehicle speed, and (2) manual and visual driver distractions induced, for example, by texting. They found a good correlation between the steering movements and the manual-visual distraction of the driver.

The above information allows one to fill, in Table 4, the relevant cells of the “Manual Distraction” column.

#### 8.1.3. Sensors

The most common solution to analyze the behavior of the driver’s hands is to use a camera placed inside the vehicle, usually near the central mirror, looking down in the direction of the driver.

Le et al. [159,160] propose an approach to detecting [159] and classifying [160] human-hand regions in a vehicle using CNNs. Their technique for hands detection is robust in difficult conditions caused, for example, by occlusions, low resolution, and/or variations of illumination.

Using deep CNNs, Yan et al. [161] classify six actions involving the driver’s hands, that is, calling, eating, smoking, keeping hands on the steering wheel, operating the gearshift, and playing on the phone. Similarly, both Baheti et al. [162] and Masood et al. [163] use ten classes to detect when the driver is engaged in activities other than safe driving, and to identify the cause of distraction.

Vehicle-based indicators can be obtained from the CAN bus of the vehicle [119,120].

The above information allows one to fill the relevant cells of Table 5.

### 8.2. State 3.2: Visual Distraction

#### 8.2.1. Description

Visual distraction occurs when the driver is looking away from the road scene, even for a split second. It is often called EOR, and is one of the most common distractions for a driver. Examples of activities causing EOR are: (1) adjusting devices in the vehicle (like a radio or navigation system); (2) looking towards other seats; (3) regarding a new message on the phone or glancing at the phone to see who is calling; and (4) looking outside when there is a distraction by the roadside. All generally result in the driver not looking straight ahead, which is what he/she needs to be doing for safe driving.

#### 8.2.2. Indicators

The gaze is the main indicator used to detect a visual distraction of a driver. The duration of EOR is probably the most-used metric. The longer the EOR duration is, the lower the SA of the driver is, and the higher the visual distraction of the driver is [164]. The glance pattern and the mean glance duration are other metrics [148].

Sometimes, the head direction is used to approximate the gaze direction in order to characterize the driver visual distraction [165,166]. For example, Fridman et al. [165] classify driver gaze regions on the sole basis of the head pose of the driver. Fridman et al. [166] compare classifications of driver gaze using either head pose alone or both head pose and eye gaze. They classify, based on facial images, the focus of the attention of the driver using 6 gaze regions (road, center stack, instrument cluster, rear-view mirror, left, and right). To do so, they consecutively perform face detection, face alignment, pupil detection, feature extraction and normalization, classification, and decision pruning. Vicente et al. [167] similarly classify the driver gaze, but use 18 regions instead of 6.

Visual distraction can also be inferred using vehicle-based indicators such as wheel steering, braking behavior, and speed. Indeed, a driver generally slows down when distracted by a visual stimulus [61,168], and visual distraction impairs lateral control because the driver needs to compensate for errors made when taking his/her eyes off the road, which leads to larger deviations in lane positioning [61,169]. Such deviations have various causes, including drowsiness and visual distraction. This re-emphasizes the need to use as many indicators as possible. This also explains why more and more vehicles are equipped with systems that keep the vehicle within its lane whenever possible.

The above information allows one to fill, in Table 4, the relevant cells of the “Visual Distraction” column.

#### 8.2.3. Sensors

In order to monitor driver visual distraction, one mainly uses at least one camera facing the driver, thus as for manual distraction. The camera can be placed in various positions as long as the head pose and/or gaze of the driver can be obtained.

Naqvi et al. [170] use a near-infrared (NIR) camera (with wavelengths of 0.75–1.4μm) placed in the dashboard in conjunction with a deep-learning-based gaze-detection system, classifying the driver gaze into 17 gaze zones.

Mukherjee and Robertson [171], similarly to Fridman et al. [165], present a CNN-based model to estimate human head pose and to classify human gaze direction. They use, however, low-resolution RGB-depth (RGB-D), thus with a camera providing depth information.

The above information allows one to fill the relevant cells of Table 5.

### 8.3. State 3.3: Auditory Distraction

#### 8.3.1. Description

Auditory distraction occurs when some sound prevents the driver from making the best use of his/her hearing, because his/her attention is drawn to the source of the sound. Hearing a phone ringing, listening to a passenger, listening to music, and following navigation instructions can all lead to auditory distraction.

This component of driver distraction is the least studied in the literature, likely because (1) it is often accompanied by at least one other more-easily detectable source of distraction falling among the other three types, and (2) it poses lower safety risks in comparison to the other types of distraction, in particular visual distraction [172].

The literature does not appear to introduce the concept of “auditory indicators”, which would characterize (1) the sounds captured both inside and outside of the vehicle, and, preferably, (2) the distraction they create. By using several microphones (including arrays thereof), and techniques for separating audio sources [173], one could imagine breaking down and localizing the various sources of sounds both inside and outside the vehicle.

#### 8.3.2. Indicators

When the driver appears to be auditorily distracted, there occur changes in pupil diameter [152,174] and blink frequency [152,175]. Brain activity (EEG) [176] can also be used as an indicator of auditory distraction. Sonnleitner et al. [177] describe the impact of an auditory secondary task on a driver during a primary driving task, and show changes in braking reaction and brain activity.

The above information allows one to fill, in Table 4, the relevant cells of the “Auditory Distraction” column.

#### 8.3.3. Sensors

As already indicated, obtaining the pupil diameter is challenging in real conditions due to illumination conditions and/or camera resolution, among others. Furthermore, brain activity cannot, at this time, be measured both in real time and in a non-intrusive, reliable way. Blink frequency can, however, be monitored via a camera, and braking behavior via the CAN bus.

Although microphones and, even better, arrays thereof, both inside and outside the vehicle, would be natural sensors to provide values for auditory indicators, we did not find any references considering such sensors for characterizing auditory distraction. One can also envision using the microphone(s) of a smartphone linked to a DMS.

The above information did not lead to the addition of any reference to Table 5.

### 8.4. State 3.4: Cognitive Distraction

#### 8.4.1. Description

In the context of driving, cognitive distraction is defined by NHTSA [178] as the mental workload associated with a task that involves thinking about something other than the (primary) driving task. A driver who is cognitively distracted due to a secondary task, such as mind wandering, experiences an increase in his/her mental workload (the state discussed in Section 7). The characterization of his/her cognitive distraction could therefore be achieved (1) by examining how his/her mental workload evolves over time and (2) by finding characteristics of this evolution allowing one to decide whether or not it is caused by cognitive distraction. The monitoring of cognitive distraction is thus, before all, a monitoring of the mental workload and/or its time variations. Section 7 shows that there are (1) many ways to characterize mental workload, and (2) many indicators thereof. The challenge is to be able to pinpoint the components of, or changes in, the mental workload that are due to distraction.

Cognitive distraction occurs when a driver is thinking about something that is not related to the driving task. In the driving context, while visual distraction can be summarized by EOR, cognitive distraction can similarly be viewed as “mind-off-road” (MOR). While it is relatively easy to monitor EOR (with a camera facing the driver), it is difficult to monitor MOR. It has, however, been shown that, when a driver is cognitively distracted, his/her visual behavior is impacted. Mind-wandering and daydreaming are two causes of cognitive distraction.

#### 8.4.2. Indicators

As cognitive distraction induces mental workload, the indicators allowing one to detect and characterize these two states are similar, if not identical. Therefore, it is difficult, if not impossible, to distinguish, in the driving context (as well as others), between these two states since they have nearly the same influences on the indicators.

Among the four types of distractions, cognitive distraction has proven to be the most difficult to detect and characterize. This is because it happens inside the brain, and, obviously, “observing” the brain of a driver is more challenging than observing his/her hands and eye(s).

As for visual distraction, cognitive distraction can be characterized by indicators of both driving performance and eye movements [141], including (1) vehicle-based indicators, such as speed [179], wheel steering [169], lane discipline [169,179,180], and braking behavior [181], and (2) driver-based, behavioral indicators, such as gaze parameters (e.g., fixation duration, glance frequency, and gaze distribution) [181,182,183,184] and head orientation. A driver makes significantly fewer high-speed saccadic eye movements and spends less time looking to the relevant periphery for impending hazards with increasing complexity of the secondary task(s). He/She also spends less time checking his/her instruments and mirrors [181].

Cognitive distraction can also be measured through a variety of driver-based, physiological indicators. Among these, brain activity [185] and pupil diameter may be the most convincing. Studies of EDA and HR show only weak relationships between these indicators and cognitive distraction [61].

Among the subjective measures, the NASA TLX [125] is commonly used in driving-distraction studies even though it is a subjective measure of mental workload, and, thus, not a measure specific to cognitive distraction.

The above information allows one to fill, in Table 4, the relevant cells of the “Cognitive Distraction” column.

#### 8.4.3. Sensors

Since the main indicators of cognitive distraction are driving performance and gaze parameters, the main sensors to characterize it are vehicle-centric sensors, and cameras.

The above information did not lead to the addition of any reference to Table 5.

## 9. State 4: Emotions

We provide a detailed description of (the state of) “emotions”, and we then present the indicators and sensors that can be used to characterize it.

### 9.1. Description

While the concept of emotions is familiar to most people, it is difficult to define. Emotions are associated with a strong feeling deriving from one’s circumstances, mood, and/or relationships with other people. In the driving context, the emotions most commonly monitored for safety purposes are stress and anger, as they have a negative impact on driving, and create dangers [186,187].

Stress is a state of physical, emotional, or psychological tension resulting from adverse or demanding circumstances. In biology, stress is defined as a state of homeostasis being challenged due to a stressor [188].

Anger is a strong feeling of annoyance, displeasure, and/or hostility. It is a common negative emotion in the context of driving, where it is often called road rage [189].

### 9.2. Indicators

Emotion recognition is currently a hot topic in the field of affective computing, and is gaining interest in the field of advanced driver-assistance systems (ADASs). To recognize emotions, one can use various behavioral features, for example, speech [190] and facial expressions [191,192].

Among the driver-based indicators of both stress and anger, physiological indicators are commonly used. Stress causes physiological responses [193], such as variations or modifications in HR [193,194,195,196], breathing activity [193,194], blood pressure, EDA [194,195,197], and pupil activity [198]. The two physiological features that exhibit the highest correlations with driver stress are HR and EDA [194].

For anger in the driving context, Wan et al. [199] suggest to identify it based on physiological indicators such as HR, EDA, breathing rate, and EEG, with the obvious, current, practical limitations for the latter.

The self-assessment manikin (SAM) [200] is a subjective assessment technique to characterize emotions.

The above information allows one to fill, in Table 4, the relevant cells of the “Emotions” column.

### 9.3. Sensors

The development of wearable devices with physiological sensors facilitates the recognition of emotions in real-driving conditions, thus outside of a laboratory context.

Facial expressions constitute a good indicator of emotions. The analysis and recognition of facial expressions is currently a field of great interest in scientific research [201,202]. Facial expressions can be monitored in a vehicle via the use of a camera facing the driver [203,204,205]. Indeed, Jeong and Ko [204] recently developed an algorithm for monitoring the emotions of a driver based on the analysis of facial expressions. Using DNNs performing facial-expression recognition (FER), they can identify—in real time and in real-driving situations—anger, disgust, fear, happiness, sadness, and surprise. A smartphone with a camera facing the user can be used for FER, here for estimating his/her emotional state [205].

Far-infrared (FIR) imaging (with wavelengths of 15–1000μm), also called infrared thermography (IRT), can be used to quantify stress and emotions by monitoring the breathing activity [206]. This can be done via the use of an IRT camera facing the driver.

The recognition of emotions can also be done using wearable sensors [207] such as the E4 wristband, which is a wearable research device that provides the means to acquire physiological data in real time. Many studies [208,209,210] have indeed shown that one can detect stress by using the physiological data that this device provides, in particular HR and EDA data.

Bořil et al. [211] developed a stress detector employing a combination of the driver’s speech and some CAN-bus parameters, mainly the steering-wheel angle and the speed. Basu et al. [212] review various methods (that are not specific to the field of driving) for recognizing emotions from speech. Zhang et al. [213] explore how to utilize a deep CNN for the same purpose.

The above information allows one to fill the relevant cells of Table 5.

## 10. State 5: Under the Influence

We provide a detailed description of (the state of) “under the influence”, and we then present the indicators and sensors that can be used to characterize it.

### 10.1. Description

Driving under the influence (DUI)—also called driving while intoxicated (DWI) and impaired driving—refers to the driving of a vehicle by a person who has consumed a quantity of alcohol or drugs (including prescription medication) that causes him/her to function in an impaired way. If the impaired driving is due only to alcohol, one also talks about drunk driving. While DUI is obviously dangerous, it is also illegal in most countries to drive under the influence of alcohol, cannabis (or marijuana), opioids, methamphetamines, and any potentially-impairing drug (e.g., a psychoactive drug), whether prescribed or over-the-counter.

A psychoactive drug, also called a psychotropic drug, is a chemical substance that changes a person’s mental state and results in alterations in perception, mood, and/or consciousness. Based on their effects, psychoactive drugs can be classified into the three main categories of stimulants, depressants, and hallucinogens [214,215]. Yet, some drugs may fall under different categories at different times (for example, cannabis is both a depressant drug and a hallucinogen drug). Stimulants (e.g., methamphetamines, cocaine) speed up the activity of the central nervous system, often resulting in the user feeling more alert, euphoric, and energetic. Depressants (e.g., heroin) slow down the activity of the central nervous system, often resulting in the user feeling more relaxed, sleepier, and insensitive to pain. Hallucinogens (e.g., LSD) are psychoactive substances that alter human sensory perceptions in such a way that the user perceives a distorted reality in which time, space, colors, and forms are altered.

The substances that are most frequently detected in impaired drivers are alcohol followed by cannabis. Studies have shown that more than one-third of adults and more than half of teenagers admit to DUI of alcohol at some point in their lives [216]. Alcohol is a depressant drug that affects the central nervous system and slows down brain functions. Any amount of alcohol can affect a person’s abilities (1) by degrading attention, perception, information processing skills, memory, reasoning, coordination, motor skills, and reaction time, and (2) by altering the five senses and the emotions [217,218,219,220]. A person’s alcohol level is measured by the weight of the alcohol in a specified volume of blood, called blood alcohol concentration (BAC) and measured in grams of alcohol per deciliter (g/dL) of blood. According to NHTSA, the effects of alcohol vary with BAC in the way shown in Table A3, in Appendix B, and the risk of having an accident after consuming alcohol increases exponentially as a function of BAC. For example, every additional 0.08 g of alcohol per deciliter (dL) of blood multiplies by four the risk of accident [216]. According to the World Health Organization [221], best practice for drunk–driving laws includes a BAC limit of 0.05 g/dL for the general population and of 0.2 g/dL for young or novice drivers. Although studies show considerable differences among individuals regarding their responses to alcohol consumption [222], young drivers experience significantly stronger effects, putting them at greater risk of accidents [223,224]. Hangovers, that is, the after-effects occurring as a result of heavy drinking and as the BAC subsequently approaches zero, are, however, known to also affect the performance of daily-life tasks, such as driving, by impairing cognitive functions, such as memory, psychomotor speed, and sustained attention [225,226].

### 10.2. Indicators

Several physiological indicators are used to monitor DUI such as heart activity [219,227], breathing activity [227], body temperature [219,228], and pupil diameter [228]. Alcohol is known to increase HR and breathing rate [227]. Cannabis is known to increase HR and breathing difficulty. Alcohol increases the activity of arteries and other blood vessels, therefore increasing the temperature of the face of a drunk person [228]. The variations of temperature are visible on the nose, eyebrows, chin, and forehead. When people drink alcohol, their irises become darker, because the sclera is replete with blood vessels that increase in temperature with alcohol consumption. In a sober person, the temperatures of the sclera and the iris are the same, but with alcohol intoxication, the temperature of the sclera increases compared to the one of the iris because of the denser blood-vessel network in the sclera.

Behavioral indicators of DUI include parameters of gaze (due to the impairment of some visual functions) and of slurred speech [227]. Drunk speakers may use prosodic contours differently from sober speakers, using more or less speech emphasis. Drunk speakers may pronounce words differently, choose certain pronunciation variants more frequently than others, and may even select more frequently certain words, the latter affecting the phonotactic patterns [229].

NHTSA [230] defines four categories of cues to predict that a driver is DUI, namely problems in (1) maintaining proper lane position (e.g., weaving, drifting, swerving), (2) controlling speed and brakes (e.g., varying speed, abnormally driving at low speed, stopping beyond a limit line), (3) maintaining vigilance (e.g., driving erroneously in opposing lanes, responding slowly to traffic signals), and (4) exercising proper judgment (e.g., following too closely, turning illegally). In congruence with the indication by NHTSA that a drunk driver is prone to weaving, drifting, and swerving (and thus to having difficulty keeping his/her vehicle in the center of the lane), an increase in SDLP is recognized in the literature to be an indicator of DUI of alcohol [231,232,233] and hangovers [226]. Speed and acceleration are other indicators, as drunk drivers often experience difficulty in keeping an appropriate speed, with abrupt accelerations or decelerations, erratic brakings, and jerky stops [231,233].

The above information allows one to fill, in Table 4, the relevant cells of the “Under the Influence” column.

### 10.3. Sensors

In police operations, alcohol levels are typically measured with a breathalyzer using air exhaled through the mouth. The amount of alcohol in breath can then be used to determine the BAC [227]. If this BAC is above the legally authorized value, the results can, if desired, be confirmed by a blood test. With just 100 microliter (μL) of collected blood, one can not only measure the BAC precisely, but also identify and quantify 37 substances that are of interest in the context of drug-impaired driving [234]. Many people, however, drive under the influence without necessarily being stopped and checked by police every time they do so.

To solve the issue of DUI, the literature commonly suggests the use of ignition-interlock devices [218,235,236]. When a driver enters his/her vehicle, he/she must provide a breath sample, and an alcohol sensor then determines whether he/she is drunk (that is, has a BAC above a specified threshold). If this is the case, the ignition-control system prevents the driver from starting the engine. Ignition-interlock devices are usually installed in the vehicles of people with prior DUI convictions and in long-haul, commercial vehicles, for example, trucks and buses [216]. This solution does not, however, allow for the real-time monitoring of the state of the driver, and does not prevent the driver from drinking alcohol after starting the engine.

To counter this problem, Sakairi [237] developed a system using a water-cluster-detecting (WCD) breath sensor that can detect breath from a distance of about 0.5 m, allowing one to monitor the driver’s alcohol level while he/she is operating his/her vehicle. The sensor detects breath by separating positively-charged water clusters in breath from negatively-charged ones by using an electric field and by measuring the two corresponding electric currents.

The detection of individuals DUI of alcohol can also be achieved based on the heart activity. Indeed, Kojima et al. [238] and Murata et al. [239] constructed a seat incorporating an air-pack sensor that monitors, via a body-trunk plethysmogram, both the heart activity and the breathing activity. The analysis, during 5min, of the extracted body-trunk plethysmogram signal, called the air-pack pulse wave, reveals differences due to the consumption of alcohol, allowing one to distinguish between sobriety and intoxication. Wu et al. [240,241] propose to use a wearable ECG sensor, and an SVM to classify the corresponding ECG data as sober or intoxicated.

Recognizing whether drivers are DUI of alcohol can also be achieved using a camera that acquires IR images [242,243,244]. For an intoxicated person, vessels on the forehead become more active so that, in an IR image, the intensities of the pixels in this region are affected accordingly. Menon et al. [244] developed a system that uses IR images of the driver’s face in order to classify him/her as sober or drunk. The system successively (1) locates the face using a CNN, and (2) performs the binary classification based on differences in blood temperatures at 22 points on the face of the driver using a supervised-learning-classification algorithm based on a probabilistic model called Gaussian-mixture model.

Rosero-Montalvo et al. [228] introduce a non-invasive system incorporating a gas sensor, a temperature sensor, and a camera to identify a person having alcohol in the blood, through supervised classification of the data from (1) the two sensors and (2) the results of the analysis of the camera output via computer vision. The authors use the concentration of alcohol in the vehicle environment, the facial temperature of the driver, and the diameters of his/her pupils.

According to NHTSA and its four, above-mentioned cues that a driver is DUI, vehicle-based indicators and related vehicle-centric sensors are of interest. Relevant CAN-bus parameters, and indicators such as wheel steering and lane discipline, are widely used to detect instances of DUI [245,246,247,248,249,250]. Harkous et al. [247] identify drunk-driving behaviors using HMMs based on vehicle-sensors data, available via the CAN bus. They use wheel-steering parameters, speed, and lateral position as indicators. They found that longitudinal-acceleration sensors achieve the best average classification accuracy for distinguishing between sobriety and intoxication. Harkous and Artail [248] extend the above work by replacing each HMM by a recurrent neural network (RNN). Likewise, Berri and Osório [245] use features such as speed, acceleration, braking, steering wheel angle, distance to the center lane, and geometry of the road (straight or curved) to detect DUI of alcohol. Their system can also be used to detect the presence of any psychoactive drug that can cause a driver to have abnormal driving behaviors. To detect an intoxicated driver, Dai et al. [251] describe a solution that only requires a mobile phone placed in the vehicle. Using the phone’s accelerometers, they analyze the longitudinal and lateral accelerations of the vehicle to detect any abnormal or dangerous driving maneuvers typically related to DUI of alcohol.

The above information allows one to fill the relevant cells of Table 5.

**Table 4:** Detailed “states vs. indicators” table, introduced in simplified form in Figure 3. Each cell in the heart of the table gives some references (if any) discussing how the corresponding indicator is useful for characterizing the corresponding state.

				States							
				Drowsiness	Mental Workload	Distraction				Emotions	Under the Influence
						Manual	Visual	Auditory	Cognitive		
**Indicators**	**Driver**	**Physiological**	**Heart Activity**	[75,76,102]	[132,133,134,135]				[61]	[193,194,195,196,199]	[219,227]
			**Breathing Activity**	[77,102]						[193,194,199]	[227]
			**Brain Activity**	[90]	[136]			[176,177]	[185]	[199]	
			**Electrodermal Activity**	[78]	[131]				[61]	[194,195,197,199]	
			**Body Temperature**								[219,228]
			**Pupil Diameter**	[79,80,81]	[33,132,137,138,139]			[152,174]		[198]	[228]
		**Behavioral**	**Gaze Parameters**	[123]	[33,140,141,142,145]		[148,164,166,167]		[181,182,183,184]		[227]
			**Blink Dynamics**	[83,87,88,89,102,123]	[33]			[152,175]		[198]	
			**PERCLOS**	[84,85,86,102,123]	[33]						
			**Facial Expressions**	[123]						[191,192]	
			**Body Posture**	[102,123]			[165,166]				
			**Hands Parameters**			[157]					
			**Speech**							[190,211]	[227,229]
		**Subjective**		[90,91,92]	[125]				[125]	[200]	
	**Vehicle**	**Wheel Steering**		[97,101,102]	[130]	[158]	[158]		[169]	[211]	
		**Lane Discipline**		[96,97,99,102,123]	[130]		[61,169]		[169,179,180]		[226,231,232,233]
		**Braking Behavior**						[177]	[181]		
		**Speed**		[100,102]			[61,168]		[179]	[211]	[231,233]
	**Environment**	**Road Geometry**			[143]						
		**Traffic Signs**									
		**Road Work**									
		**Traffic Density**			[143,146]						
		**Obstacles**			[143]						
		**Weather**									

**Table 5:** Detailed “sensors vs. indicators” table, introduced in simplified form in Figure 3. Each cell in the heart of the table gives some references (if any) discussing how the corresponding sensor is useful for characterizing the corresponding indicator. The indicators are identical to the ones in Table 4, thereby allowing one to link both tables.

				Sensors								
				Driver						Vehicle	Environment	
				Seat	Steering Wheel	Safety Belt	Internal Camera	Internal Microphone	Wearable	CAN Bus	External Camera	Radar
**Indicators**	**Driver**	**Physiological**	**Heart Activity**	[106,107,238,239]	[105]	[109]	[108]		[208,209,210,240,241]			
			**Breathing Activity**				[77,206]					
			**Brain Activity**									
			**Electrodermal Activity**						[208,209,210]			
			**Body Temperature**				[242,243,244]					
			**Pupil Diameter**				[139,228]					
		**Behavioral**	**Gaze Parameters**				[116,123,140,144,145,166,167,170,171]					
			**Blink Dynamics**				[104,112,114,116,117,118,123]					
			**PERCLOS**				[113,116,123]					
			**Facial Expressions**				[123,203,204,205]					
			**Body Posture**	[115]			[114,116,117,118,123]					
			**Hands Parameters**				[159,160,161,162,163]					
			**Speech**					[190,211,212,213]				
		**Subjective**										
	**Vehicle**	**Wheel Steering**								[119,120,245,247,248,249,250]		
		**Lane Discipline**								[245,247,248,250]	[122,123]	
		**Braking Behavior**								[119,120,245]		
		**Speed**								[119,120,245,247,248]		
	**Environment**	**Road Geometry**									[143]	
		**Traffic Signs**										
		**Road Work**										
		**Traffic Density**										
		**Obstacles**										[252]
		**Weather**										

## 11. Summary and Conclusions

This paper focuses on the characterization of the state of a driver, which is the first key step for driver monitoring (DM) and driver monitoring systems (DMSs). It surveys (in Section 3) the relevant scientific and technical literature on driver-state characterization, and subsequently provides a synthesis (in Section 4, Section 5, Section 6, Section 7, Section 8, Section 9 and Section 10) of the main, published techniques for this characterization.

The survey yielded 56 publications in scientific/technical journals and conference proceedings. Their examination led to the conclusion that the state of a driver should be characterized according to five main dimensions—called here “(sub)states ”—of drowsiness, mental workload, distraction (further subdivided into four types qualified of manual, visual, auditory, and cognitive), emotions, and under the influence.

In comparison with standard physical quantities, such as voltage and power, these states are not well defined and/or are very difficult—if at all possible—to quantify or to label, not only in a validated way, but also in real time and non-invasively, as is required in the driving context. The only reasonable approach, found almost universally in the literature, is to have recourse to indicators (of each of these states), the value of which can be obtained in a practical and validated way. Examples of indicators are the eye-blink rate, the standard deviation of lane departure (SDLP), and the outside temperature. The values of many indicators (but not all) are obtained by applying algorithms, often complex, to data (typically signals and images) collected from sensors.

The last paragraph brings to light the three ingredients that, in our view, lie at the heart of DM and DMSs, that is, the triad of states, indicators (of these states), and sensors (providing data, which are the source of the values of these indicators). Figure 2 links these three ingredients.

Our survey confirmed the intuition that one should monitor, not only the driver (D), but also the (driven) vehicle (V) and the (driving) environment (E). Accordingly, we partitioned both the indicators and the sensors into D, V, and E categories, leading to the phrases “X-based indicators” and “X-centric sensors”, where X can be D, V, or E. For the D-based indicators, we further distinguished between three types: physiological, behavioral, and subjective. The three examples of indicators given earlier correspond to D, V, and E, respectively.

The major outcome of the paper is the pair of interlocked tables “states vs indicators” (Table 4) and “sensors vs indicators” (Table 5), where each cell contains zero, one, or more references. These tables bring together, in an organized way, most of the useful information found in the literature, up to the time of this writing, about driver-state characterization for DM and DMSs. These tables constitute an up-to-date, at-a-glance, visual reference guide for anyone active in this field. They provide immediate answers to key questions that arise in the design of DMSs, such as the four questions posed in Section 5.

The pair of tables and the references they contain lead to the following main conclusions:

1. Each state can be inferred from several indicators (which are often far from perfect), thereby encouraging multimodal fusion.
2. The internal camera (possibly with several instances) appears to be the most-commonly-used sensor.
3. Wearable sensors (e.g., smartwatches) are increasingly used to obtain driver-based, physiological indicators and vehicle-based indicators.
4. Environment-based indicators are often ignored, even though there is an agreement that they should be used.
5. Driver-based, subjective indicators, although sometimes alluded to, cannot be used in real driving but are essential for the validation of some indicators of some states.
6. Brain activity is a recognized indicator of several states, but cannot be accessed today in a non-invasive, reliable, and inexpensive way in real driving.
7. Several methods for characterizing each of the five states use, without surprise, techniques of machine learning (ML) and, especially, of deep learning.
8. The term “predict(ion)” often refers to a present state rather than to a future state, and few papers describe techniques “to tell beforehand”, for example, the future values of indicators and levels of states.

The next two paragraphs respectively elaborate on the last two points.

For driving safety, it is paramount that the processing and decisions made by any algorithm used in a vehicle, including for DM, be fully explainable (to a human being) at the time of design and certification of this algorithm. Most algorithms using ML do not, however, have this necessary feature of explainability or interpretability, and this is certainly the case for ML-based algorithms that would learn on-the-fly during one or more trips. Therefore, while ML algorithms and, especially, deep-learning algorithms often provide stellar performances on specific datasets in comparison with other types of algorithms, they will almost certainly not be acceptable to an equipment provider or a vehicle manufacturer. There is, however, a trend toward designing ML algorithms that produce results that can be explained [253,254]. The above remarks apply not only to ML but also to any approach whose operation cannot be explained simply. Our framework, which implies the use of indicators and states, supports the desired explainability. It indeed prevents any algorithm from going, in one fell swoop, from (nearly-)raw sensor data to driver characterization, by forcing it to estimate both the values of indicators and the levels of states as a stepping stone toward the ultimate characterization of the state of a driver.

The literature on DM focuses almost exclusively on characterizing the “present” state of the driver. We use quotes because the characterization is typically based on data from the recent past, for example, in a window that extends over several tens of seconds and butts against almost the present time. This results in a characterization of the “recent-past” state of the driver. If the driver is in control, a DMS using this characterization may not have sufficient lead time to take proper emergency action (to issue an alarm and/or to take back the control) and, if the vehicle is in control, such a DMS may hand the control over to the driver even though he/she might be falling asleep or getting distracted in a few tens of seconds or more. A major missing link in current DMS research and development is thus the true prediction of the future state of the driver, at least a few tens of seconds into the future.

On the one hand, Table 4 and Table 5 show, at a glance, which areas of driver-state characterization have been the object of research and with what intensity (as measured by the number of references listed in each cell). For example, Table 4 shows that significant research has been performed to analyze the emotions of the driver using the driver-based, physiological indicators of heart rate, breathing activity, and electrodermal activity. On the other hand, the two tables show, also at a glance, where little or no research has been performed to date, thereby suggesting new, potentially-fruitful research areas. The two tables should thus prove to be a rich source of information for both research and product development.

**Figure A1:**
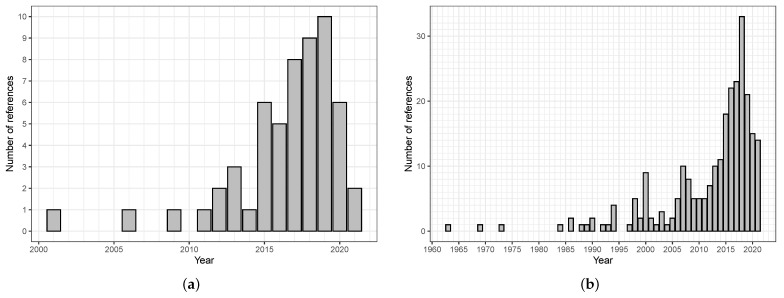
Graph (**a**) is the histogram of the number of references vs. year for the 56 initial references on driver monitoring (DM), and graph (**b**) is the corresponding histogram for the 254 examined references. These histograms suggest that the field of DM has been the object of growing interest over the years and, in particular, over the last 10 years.

Starting from a set of 56 initial references, our exploration of the field of DM led us to examine a total of 254 references. While our criss-crossing of the field, at several different times, led us to identify many relevant publications, our search cannot, obviously, be exhaustive. In any case, the two histograms of “number of references vs year” of Figure A1 (for the 56 and 254 references, respectively) constitute a clue that the research activity in DM has been accelerating over the past decade.

The methodology used in this paper can be applied to update the tables at various times in the future to take into account new developments. This can be done by adding and/or removing rows, columns, and/or references, as appropriate.

Characterizing the state of a driver and, more generally, DM will remain important despite the progressive increase in vehicle automation. SAE Level 3 enables vehicles to drive by themselves under certain conditions such as on a highway and in sunny weather, but a driver must still be present and able to take back control of the vehicle at any time and in a relatively short lapse of time. In order to ensure that the driver is able to take back control, technologies for monitoring the state of the driver will become even more critical. These technologies are also needed to monitor the driver during the time he/she is driving, and to possibly allow the vehicle to take back control if necessary.

Currently, some vehicle manufacturers offer DMSs based on the behavior of the driver and/or the behavior of the vehicle, such as the detection of steering-wheel movements and lane deviations, respectively. These systems can be useful in current vehicles with automation up to (SAE) Level 2, but will become obsolete at higher levels of automation. Indeed, when a vehicle drives autonomously, monitoring its behavior does not provide any information about the state of the driver, and technologies that directly monitor both the driver and the driving environment are a necessity as long as the driver is involved in the driving task, at least partially.

To date, the development of driving-automation systems (DASs) has moved at a faster pace than has the development of DMSs. This is, in major part, a consequence of the long-held belief by some automotive-industry players that they would be able to easily leapfrog Levels 3 and 4, and move on directly to Level 5, where there is no need to monitor the driver. However, most experts now agree that it will be decades before most privately-owned vehicles are fully automated, if ever. Along the long and winding road to Level 5, the automotive industry will need to significantly boost the research on, and the development of, DMSs. For Levels 3 and 4, the same industry will need to develop automated-driving systems (ADSs) and DMSs in full synergy. The future could thus not be brighter for the field of DM and DMSs.

NASA TLX

## Figures and Tables

**Table 1:** This table shows the role played by each of the four key actors, that is, driver, driver-support (DS) features, automated-driving (AD) features, and driver monitoring (DM), at each of the six SAE Levels of driving automation (from 0 to 5).

	SAE Levels	0	1	2	3	4	5
Actors		No Driving Automation	Driver Assistance	Partial Driving Automation	Conditional Driving Automation	High Driving Automation	Full Driving Automation
**Driver**		Driving and supervising DS features			Driving when AD features request it	Driving (if desired) when AD features reach their limits	/
**Driver-Support (DS)** **Features**		Warning and temporary support	Lateral or longitudinal support	Lateral and longitudinal support	/	/	/
**Automated-** **Driving (AD) Features**		/	/	/	Driving when AD features permit it		Driving
**Driver** **Monitoring** **(DM)**		Monitoring	Monitoring with relevant indicators		Monitoring fallback- ready driver	Monitoring when driver in control	/

**Table 2:** The first column of the table lists, by alphabetical order of first author, the 56 references that resulted from our survey on driver monitoring (DM) and related systems (DMSs). The next three megacolumns and the last column briefly describe, for each reference, the states, indicators, sensors, and test conditions considered therein.

References	States					Indicators					Sensors			Tests
	Drowsiness	Mental Workload	Distraction	Emotions	Under the Influence	Driver			Vehicle	Environment	Driver	Vehicle	Environment	
						Physiological	Behavioral	Subjective						
Ahir and Gohokar [8]	V					HR, brain	gaze, blink, PERCLOS, facial, body		wheel, lane, speed		cam, elec		ext cam	real, sim
Alluhaibi et al. [9]	V		V	ang			speech		wheel, lane, brake, speed		cam *, mic *	V *		
Arun et al. [10]			vis, cog			HR, brain, EDA, pupil	gaze, blink, body	V	wheel, lane, brake, speed		cam, wea d, eye t	V		sim
Balandong et al. [11]	V					HR, brain	gaze, blink, PERCLOS, body	V	wheel, lane, brake, speed		elec			sim
Begum [12]	V		V	stress		HR, brain					seat, ste w, saf b, wea d			real, sim
Chacon-Murguia and Prieto-Resendiz [13]	V					HR, brain, EDA	gaze, blink, body		wheel, lane, brake, speed		ste w, cam		radar	real
Chan et al. [14]	V					HR, brain	blink, PERCLOS, facial, body		wheel, brake, speed		cam *, mic *			real
Chhabra et al. [15]	V		V		alc	breath	gaze, PERCLOS, facial, body		wheel	road	seat, cam *, mic *	V *		real, sim
Chowdhury et al. [16]	V					HR, brain, EDA	blink, PERCLOS							sim
Chung et al. [17]				stress		HR, breath, brain, EDA, pupil	speech	V	wheel, lane, brake, speed		cam, wea d	V		real, sim
Coetzer and Hancke [18]	V					brain	gaze, PERCLOS, facial, body		wheel, lane, speed		cam	V		real, sim
Dababneh and El-Gindy [19]	V					brain, EDA, pupil	blink, PERCLOS, body		wheel, lane, speed	road	cam, wea d		radar	real, sim
Dahiphale and Rao [20]	V		V				gaze, blink, facial, body		wheel		cam			real
Dong et al. [21]	V		V			HR, brain, pupil	gaze, blink, PERCLOS, facial, body	V	wheel, lane, speed	road, wea	cam	V		real
El Khatib et al. [5]	V		man, vis, cog			HR, breath, brain, EDA, pupil	gaze, blink, PERCLOS, facial, body, hands		wheel, lane, speed		cam	V *	ext cam, radar	real, sim
Ghandour et al. [22]			man, vis, aud, cog	stress		HR, breath, brain, EDA	gaze, facial, body, speech	V	wheel, brake, speed		cam, wea d			real, sim
Hecht et al. [23]	V	V	V			HR, brain, EDA, pupil	gaze, blink, PERCLOS, facial, body	V			elec, eye t			real, sim
Kang [24]	V		V			HR, breath, brain, EDA	gaze, blink, facial, body		wheel, lane, brake, speed		seat, ste w, cam	V		real, sim
Kaplan et al. [25]	V		V			HR, brain	gaze, blink, PERCLOS, facial, body, speech		wheel, lane, brake, speed		ste w, cam *, mic *, wea d	V		real, sim
Kaye et al. [26]	V			stress		HR, breath, brain, EDA		V						real, sim
Khan and Lee [27]	V		man, vis, aud, cog			HR, brain, EDA	gaze, PERCLOS, body		wheel, lane, brake, speed		wea d			real
Kumari and Kumar [28]	V					HR, brain	gaze, blink, PERCLOS, body	V	wheel, lane		cam			
Lal and Craig [29]	V					HR, brain, EDA	PERCLOS, facial				cam			sim
Laouz et al. [30]	V					HR, brain, EDA	blink, PERCLOS, facial, body	V	wheel, speed		seat, cam, wea d		ext cam	real
Leonhardt et al. [31]						HR, breath					seat, ste w, saf b, cam			real
Liu et al. [32]	V					HR, brain, pupil	gaze, blink, PERCLOS, body		wheel, lane, speed		cam	V		real
Marquart et al. [33]		V				pupil	gaze, blink, PERCLOS	V			eye t			real, sim
Marina Martinez et al. [34]				ang					brake, speed			V *		
Mashko [35]	V					HR, brain, EDA	gaze, blink, body		wheel, lane, brake, speed		cam, wea d	V	ext cam, radar	real, sim
Mashru and Gandhi [36]	V					HR, breath	blink, PERCLOS, facial, body	V	wheel, lane		seat, ste w, cam, wea d			sim
Melnicuk et al. [37]	V	V	cog	stress, ang		HR, brain	blink, PERCLOS, facial		wheel, brake, speed	road, traf, wea	seat, ste w, saf b, cam *, wea d	V *		real
Mittal et al. [38]	V					HR, brain, pupil	blink, PERCLOS, body	V	wheel, lane, brake, speed		cam, elec	V	ext cam	real
Murugan et al. [39]	V					HR, breath, brain, EDA, pupil	blink, PERCLOS, body	V	wheel, lane, speed		cam, elec	V		sim
Nair et al. [40]	V		V		alc		gaze, PERCLOS, facial, body		lane		seat, cam *	V	radar	
Němcová et al. [41]	V			stress		HR, breath, brain, EDA	gaze, blink, PERCLOS, facial, body		wheel, lane, brake, speed		seat, ste w, cam, wea d, eye t	V		real, sim
Ngxande et al. [42]	V						blink, PERCLOS, facial, body				cam			
Oviedo-Trespalacios et al. [43]		V	V				gaze		wheel, lane, brake, speed					real, sim
Papantoniou et al. [44]		V	V			HR, breath, brain	gaze, blink, speech	V	wheel, lane, speed		cam		ext cam, radar	real, sim
Pratama et al. [45]	V					HR, brain, EDA	gaze, blink, PERCLOS, facial, body, hands	V	wheel, lane		cam, wea d, elec		ext cam	real, sim
Ramzan et al. [46]	V					HR, breath, brain	blink, PERCLOS, facial, body		wheel, lane, speed		cam, wea d, elec	V		real, sim
Sahayadhas et al. [47]	V					HR, brain, pupil	gaze, blink, PERCLOS, body	V	wheel, lane		seat, ste w, cam, wea d	V		real, sim
Scott-Parker [48]				stress, ang		HR, brain, EDA	gaze, facial	V	wheel, lane, brake, speed	traf	eye t		ext cam	real, sim
Seth [49]	V										cam	V		real
Shameen et al. [50]	V					brain	gaze, blink				elec			sim
Sigari et al. [51]	V						gaze, blink, PERCLOS, facial, body				cam			real
Sikander and Anwar [52]	V					HR, brain, pupil	gaze, blink, PERCLOS, body	V	wheel, lane		seat, ste w, saf b, cam, wea d, elec			real
Singh and Kathuria [53]	V	V	V	V		pupil	gaze, blink, PERCLOS, facial		wheel, brake, speed	road, traf	cam, wea d	V	ext cam, radar	real
Subbaiah et al. [54]	V					HR, brain, pupil	blink, PERCLOS, facial, body				cam			real, sim
Tu et al. [55]	V					HR, brain	blink, PERCLOS, facial, body		wheel, lane, speed		cam *, wea d, elec	V		real, sim
Ukwuoma and Bo [56]	V					HR, breath, brain	blink, PERCLOS, facial, body		wheel, lane, brake		cam, wea d, elec			real
Vilaca et al. [57]	V		V			brain	gaze, body		wheel, lane, brake, speed		cam, mic	V	ext cam	
Vismaya and Saritha [58]			V				gaze, blink, PERCLOS, body				cam, eye t			real, sim
Wang et al. [59]	V					brain, pupil	gaze, blink, PERCLOS, body		lane		cam, wea d			real, sim
Welch et al. [60]				stress, ang		HR, breath, brain, EDA	blink, facial, speech		wheel, brake, speed		seat, ste w, cam, wea d	V		real, sim
Yusoff et al. [61]			vis, cog			HR, brain, EDA, pupil	gaze, body	V	lane, speed		eye t			
Zhang et al. [62]	V					HR, brain	gaze, blink, PERCLOS, body		lane, speed		cam		ext cam	real, sim

**Table 3:** The table defines the abbreviations used in Table 2. They are organized according to the megacolumns and columns of Table 2, and are listed in alphabetical order.

States		Indicators		Sensors		Tests	
***Distraction***		***Driver***		***Driver***		real	real conditions
aud	auditory	blink	blink dynamics	cam	camera	sim	simulated conditions
cog	cognitive	body	body posture	elec	electrode(s)		
man	manual	brain	brain activity	eye t	eye tracker		
vis	visual	breath	breathing activity	mic	microphone		
***Emotions***		EDA	electrodermal activity	saf b	safety belt		
ang	anger	facial	facial expressions	ste w	steering wheel		
***Under the Influence***		hands	hands parameters	***Environment***			
alc	alcohol	HR	heart rate/activity	ext cam	external camera		
		pupil	pupil diameter				
		***Vehicle***					
		brake	braking behavior				
		lane	lane discipline				
		wheel	wheel steering				
		***Environment***					
		road	road geometry				
		traf	traffic density				
		wea	weather				

## Appendix A. Printable Version of Table of 56 Initial References

Table A1 and Table A2 constitute a version of Table 2 suitable for printing.

**Table A1:** This table gives the three main columns of Table 2 labelled “States”, “Sensors”, and “Tests”. The remaining main column “Indicators” is provided in Table A2. This partitioning of Table 2 allows for more comfortable visualization of its content when printed.

References	States					Sensors			Tests
	Drowsiness	Mental Workload	Distraction	>Emotions	Under the Influence	Driver	Vehicle	Environment	
Ahir and Gohokar [8]	V					cam *, mic *		ext cam	real, sim
Alluhaibi et al. [9]	V		V	ang		cam *, mic *	V *		
Arun et al. [10]			vis, cog			cam, wea d, eye t	V		sim
Balandong et al. [11]	V					elec			sim
Begum [12]	V		V	stress		seat, ste w, saf b, wea d			real, sim
Chacon-Murguia and Prieto-Resendiz [13]	V					ste w, cam		radar	real
Chan et al. [14]	V					cam *, mic *			real
Chhabra et al. [15]	V		V		alc	seat, cam *, mic *	V *		real, sim
Chowdhury et al. [16]	V								sim
Chung et al. [17]				stress		cam, wea d	V		real, sim
Coetzer and Hancke [18]	V					cam	V		real, sim
Dababneh and El-Gindy [19]	V					cam, wea d		radar	real, sim
Dahiphale and Rao [20]	V		V			cam			real
Dong et al. [21]	V		V			cam	V		real
El Khatib et al. [5]	V		man, vis, cog			cam	V *	ext cam, radar	real, sim
Ghandour et al. [22]			man, vis, aud, cog	stress		cam, wea d			real, sim
Hecht et al. [23]	V	V	V			elec, eye t			real, sim
Kang [24]	V		V			seat, ste w, cam	V		real, sim
Kaplan et al. [25]	V		V			ste w, cam *, mic *, wea d	V		real, sim
Kaye et al. [26]	V			stress					real, sim
Khan and Lee [27]	V		man, vis, aud, cog			wea d			real
Kumari and Kumar [28]	V					cam			
Lal and Craig [29]	V					cam			sim
Laouz et al. [30]	V					seat, cam, wea d		ext cam	real
Leonhardt et al. [31]						seat, ste w, saf b, cam			real
Liu et al. [32]	V					cam	V		real
Marquart et al. [33]		V				eye t			real, sim
Marina Martinez et al. [34]				ang			V *		
Mashko [35]	V					cam, wea d	V	ext cam, radar	real, sim
Mashru and Gandhi [36]	V					seat, ste w, cam, wea d			sim
Melnicuk et al. [37]	V	V	cog	stress, ang		seat, ste w, saf b, cam *, wea d	V *		real
Mittal et al. [38]	V					cam, elec	V	ext cam	real
Murugan et al. [39]	V					cam, elec	V		sim
Nair et al. [40]	V		V		alc	seat, cam *	V	radar	
Němcová et al. [41]	V			stress		seat, ste w, cam, wea d, eye t	V		real,sim
Ngxande et al. [42]	V					cam			
Oviedo-Trespalacios et al. [43]		V	V						real, sim
Papantoniou et al. [44]		V	V			cam		ext cam, radar	real, sim
Pratama et al. [45]	V					cam, wea d, elec		ext cam	real, sim
Ramzan et al. [46]	V					cam, wea d, elec	V		real, sim
Sahayadhas et al. [47]	V					seat, ste w, cam, wea d	V		real, sim
Scott-Parker [48]				stress, ang		eye t		ext cam	real, sim
Seth [49]	V					cam	V		real
Shameen et al. [50]	V					elec			sim
Sigari et al. [51]	V					cam			real
Sikander and Anwar [52]	V					seat, ste w, saf b, cam, wea d, elec			real
Singh and Kathuria [53]	V	V	V	V		cam, wea d	V	ext cam, radar	real
Subbaiah et al. [54]	V					cam			real, sim
Tu et al. [55]	V					cam *, wea d, elec	V		real, sim
Ukwuoma and Bo [56]	V					cam, wea d, elec			real
Vilaca et al. [57]	V		V			cam, mic	V	ext cam	
Vismaya and Saritha [58]			V			cam, eye t			real, sim
Wang et al. [59]	V					cam, wea d			real, sim
Welch et al. [60]				stress, ang		seat, ste w, cam, wea d	V		real, sim
Yusoff et al. [61]			vis, cog			eye t			
Zhang et al. [62]	V					cam		ext cam	real, sim

**Table A2:** This table gives the main column of Table 2 labelled “Indicators”. The three remaining main columns “States”, “Sensors”, and “Tests” are provided in Table A1. This partitioning of Table 2 allows for more comfortable visualization of its content when printed.

References	Indicators				
	Driver			Vehicle	Environment
	Physiological	Behavioral	Subjective		
Ahir and Gohokar [8]	HR, brain	gaze, blink, PERCLOS, facial, body		wheel, lane, speed	
Alluhaibi et al. [9]		speech		wheel, lane, brake, speed	
Arun et al. [10]	HR, brain, EDA, pupil	gaze, blink, body	V	wheel, lane, brake, speed	
Balandong et al. [11]	HR, brain	gaze, blink, PERCLOS, body	V	wheel, lane, brake, speed	
Begum [12]	HR, brain				
Chacon-Murguia and Prieto-Resendiz [13]	HR, brain, EDA	gaze, blink, body		wheel, lane, brake, speed	
Chan et al. [14]	HR, brain	blink, PERCLOS, facial, body		wheel, brake, speed	
Chhabra et al. [15]	breath	gaze, PERCLOS, facial, body		wheel	road
Chowdhury et al. [16]	HR, brain, EDA	blink, PERCLOS			
Chung et al. [17]	HR, breath, brain, EDA, pupil	speech	V	wheel, lane, brake, speed	
Coetzer and Hancke [18]	brain	gaze, PERCLOS, facial, body		wheel, lane, speed	
Dababneh and El-Gindy [19]	brain, EDA, pupil	blink, PERCLOS, body		wheel, lane, speed	road
Dahiphale and Rao [20]		gaze, blink, facial, body		wheel	
Dong et al. [21]	HR, brain, pupil	gaze, blink, PERCLOS, facial, body	V	wheel, lane, speed	road, wea
El Khatib et al. [5]	HR, breath, brain, EDA, pupil	gaze, blink, PERCLOS, facial, body, hands		wheel, lane, speed	
Ghandour et al. [22]	HR, breath, brain, EDA	gaze, facial, body, speech	V	wheel, brake, speed	
Hecht et al. [23]	HR, brain, EDA, pupil	gaze, blink, PERCLOS, facial, body	V		
Kang [24]	HR, breath, brain, EDA	gaze, blink, facial, body		wheel, lane, brake, speed	
Kaplan et al. [25]	HR, brain	gaze, blink, PERCLOS, facial, body, speech		wheel, lane, brake, speed	
Kaye et al. [26]	HR, breath, brain, EDA		V		
Khan and Lee [27]	HR, brain, EDA	gaze, PERCLOS, body		wheel, lane, brake, speed	
Kumari and Kumar [28]	HR, brain	gaze, blink, PERCLOS, body	V	wheel, lane	
Lal and Craig [29]	HR, brain, EDA	PERCLOS, facial			
Laouz et al. [30]	HR, brain, EDA	blink, PERCLOS, facial, body	V	wheel, speed	
Leonhardt et al. [31]	HR, breath				
Liu et al. [32]	HR, brain, pupil	gaze, blink, PERCLOS, body		wheel, lane, speed	
Marquart et al. [33]	pupil	gaze, blink, PERCLOS	V		
Marina Martinez et al. [34]				brake, speed	
Mashko [35]	HR, brain, EDA	gaze, blink, body		wheel, lane, brake, speed	
Mashru and Gandhi [36]	HR, breath	blink, PERCLOS, facial, body	V	wheel, lane	
Melnicuk et al. [37]	HR, brain	blink, PERCLOS, facial		wheel, brake, speed	road, traf, wea
Mittal et al. [38]	HR, brain, pupil	blink, PERCLOS, body	V	wheel, lane, brake, speed	
Murugan et al. [39]	HR, breath, brain, EDA, pupil	blink, PERCLOS, body	V	wheel, lane, speed	
Nair et al. [40]		gaze, PERCLOS, facial, body		lane	
Němcová et al. [41]	HR, breath, brain, EDA	gaze, blink, PERCLOS, facial, body		wheel, lane, brake, speed	
Ngxande et al. [42]		blink, PERCLOS, facial, body			
Oviedo-Trespalacios et al. [43]		gaze		wheel, lane, brake, speed	
Papantoniou et al. [44]	HR, breath, brain	gaze, blink, speech	V	wheel, lane, speed	
Pratama et al. [45]	HR, brain, EDA	gaze, blink, PERCLOS, facial, body, hands	V	wheel, lane	
Ramzan et al. [46]	HR, breath, brain	blink, PERCLOS, facial, body		wheel, lane, speed	
Sahayadhas et al. [47]	HR, brain, pupil	gaze, blink, PERCLOS, body	V	wheel, lane	
Scott-Parker [48]	HR, brain, EDA	gaze, facial	V	wheel, lane, brake, speed	traf
Seth [49]					
Shameen et al. [50]	brain	gaze, blink			
Sigari et al. [51]		gaze, blink, PERCLOS, facial, body			
Sikander and Anwar [52]	HR, brain, pupil	gaze, blink, PERCLOS, body	V	wheel, lane	
Singh and Kathuria [53]	pupil	gaze, blink, PERCLOS, facial		wheel, brake, speed	road, traf
Subbaiah et al. [54]	HR, brain, pupil	blink, PERCLOS, facial, body			
Tu et al. [55]	HR, brain	blink, PERCLOS, facial, body		wheel, lane, speed	
Ukwuoma and Bo [56]	HR, breath, brain	blink, PERCLOS, facial, body		wheel, lane, brake	
Vilaca et al. [57]	brain	gaze, body		wheel, lane, brake, speed	
Vismaya and Saritha [58]		gaze, blink, PERCLOS, body			
Wang et al. [59]	brain, pupil	gaze, blink, PERCLOS, body		lane	
Welch et al. [60]	HR, breath, brain, EDA	blink, facial, speech		wheel, brake, speed	
Yusoff et al. [61]	HR, brain, EDA, pupil	gaze, body	V	lane, speed	
Zhang et al. [62]	HR, brain	gaze, blink, PERCLOS, body		lane, speed	

## Appendix B. Effects of Blood Alcohol Concentration

As of this writing (in mid 2021), the NHTSA website contains a webpage about “Drunk Driving”, which features a table entitled “The Effects of Blood Alcohol Concentration”. Table A3 reproduces this table, nearly verbatim, in compliance with the “Terms of Use” of the website. The table shows, as a function of the level of blood alcohol concentration (BAC) (in g/dL), (1) the typical effects, independently of any task, and (2) the predictable effects for the specific task of driving.

**Table A3:** This table gives the effects of blood alcohol concentration (BAC). It is a nearly-verbatim reproduction of a table present on the NHTSA website in mid 2021.

BAC (in g/dL)	Typical Effects	Predictable Effects on Driving
0.02	Some loss of judgment; relaxation, slight body warmth, altered mood	Decline in visual functions (rapid tracking of a moving target), decline in ability to perform two tasks at the same time (divided attention)
0.05	Exaggerated behavior, may have loss of small-muscle control (e.g., focusing your eyes), impaired judgment, usually good feeling, lowered alertness, release of inhibition	Reduced coordination, reduced ability to track moving objects, difficulty steering, reduced response to emergency driving situations
0.08	Muscle coordination becomes poor (e.g., balance, speech, vision, reaction time, and hearing), harder to detect danger; judgment, self-control, reasoning, and memory are impaired	Concentration, short-term memory loss, speed control, reduced information processing capability (e.g., signal detection, visual search), impaired perception
0.10	Clear deterioration of reaction time and control, slurred speech, poor coordination, and slowed thinking	Reduced ability to maintain lane position and brake appropriately
0.15	Far less muscle control than normal, vomiting may occur (unless this level is reached slowly or a person has developed a tolerance for alcohol), major loss of balance	Substantial impairment in vehicle control, attention to driving task, and in necessary visual and auditory information processing

## Appendix C. Growth of Literature on Driver Monitoring

The survey of Section 3 provided an initial set of 56 references for the field of DM. They appear in Table 2. Our overall analysis and synthesis of the field led us to examine in detail a total of 254 references, including the 56 initial ones. They all appear in the “References” section.

To characterize, in an approximate way, the evolution of the number of publications on DM over recent years, (1) we computed, for the 56 initial references, the number of them published during each of the years they cover, and (2) we did the same for the 254 examined references. Figure A1 gives the corresponding graphs, or histograms, of “number of references vs year”. Each histogram shows a significant growth over the last 10 years or so. The significant dip in 2020 could be an effect of the difficult worldwide situation during that year.
